# Statistical meta-analysis to investigate the association between the Interleukin-6 (IL-6) gene polymorphisms and cancer risk

**DOI:** 10.1371/journal.pone.0247055

**Published:** 2021-03-08

**Authors:** Md. Harun-Or-Roshid, Md. Borqat Ali, Jesmin, Md. Nurul Haque Mollah

**Affiliations:** 1 Bioinformatics Laboratory, Department of Statistics, University of Rajshahi, Rajshahi, Bangladesh; 2 Department of Genetic Engineering and Biotechnology, University of Dhaka, Dhaka, Bangladesh; Indiana University Bloomington, UNITED STATES

## Abstract

A good number of genome-wide association studies (GWAS), including meta-analyses, reported that single nucleotide polymorphisms (SNPs) of the IL-6 gene are significantly associated with various types of cancer risks, though some other studies reported insignificant association with cancers, in the literature. These contradictory results may be due to variations in sample sizes and/or deficiency of statistical modeling. Therefore, an attempt is made to provide a more comprehensive understanding of the association between the IL-6 gene SNPs (rs1800795, rs1800796, rs1800797) and different cancer risks, giving the weight on a large sample size, including different cancer types and appropriate statistical modeling with the meta-dataset. In order to attain a more reliable consensus decision about the association between the IL-6 gene polymorphisms and different cancer risks, in this study, we performed a multi-case statistical meta-analysis based on the collected information of 118 GWAS studies comprising of 50053 cases and 65204 control samples. Results from this Meta-analysis indicated a significant association (*p*-value < 0.05) of the IL-6 gene rs1800796 polymorphism with an overall increased cancer risk. The subgroup analysis data based on cancer types exhibited significant association (*p*-value < 0.05) of the rs1800795 polymorphism with an overall increased risk of cervical, liver and prostate cancers; the rs1800796 polymorphism with lung, prostate and stomach cancers; and the rs1800797 polymorphism with cervical cancer. The subgroup analysis of ethnicity data showed a significant association (*p*-value < 0.05) of an overall cancer risk with the rs1800795 polymorphism for the African and Asian populations, the rs1800796 polymorphism for the Asian only and the rs1800797 polymorphism in the African population. Comparative discussion showed that our multi-case meta-analyses received more support than any previously reported individual meta-analysis about the association between the IL-6 gene polymorphisms and cancer risks. Results from this study, more confidently showed that the IL-6 gene SNPs (rs1800795, rs1800796 and rs1800797) in humans are associated with increased cancer risks. Therefore, these three polymorphisms of the IL-6 gene have the potential to be evaluated as a population based rapid, low-cost PCR prognostic biomarkers for different types of cancers diagnosis and research.

## Introduction

Cancer is a leading cause of death worldwide. According to the World Health Organization (WHO), 9.6 million deaths occurred in 2018 from 18.1 million cancer patients all over the globe. It has been estimated that the incidence of cancer occurrences might be increased by 50% to 15 million new cases by the year 2020 [1]. The GLOBOCAN database published the extent of mortality and outbreak in 2018 from 36 types of cancer in 185 countries [2]. According to the recent literature reviews, it is very much evident now that cancer is a multi-factorial progressive disorder that developed under the influence of genes and their interactions [2–4].

Interleukin-6 (IL-6) gene encodes a cytokine that functions in inflammation and has been reported in association with cancers in the literature for many years [3, 4]. Growing evidence suggests an important role for pro-inflammatory cytokines like the IL-6 gene in the microenvironment of tumor development and regarded as an important tumor promoting factor in various types of human cancers including breast, oral, gastrointestinal, prostate, and, colorectal cancer [5–12]. The IL-6 rs1800795 (-174G/C) polymorphism is a significant predictor for susceptibility of prostate cancer and bone metastasis in northwest Iranian population [11]. A number of meta-analysis of the IL-6 gene polymorphisms with cancer risk were conducted based on small sample sizes [123–130]. Some GWA studies have also reported that some polymorphisms of the IL-6 gene are insignificantly associated with blood cancer [13–25] and significantly associated with breast cancer [26–36] whereas some other studies [37–40] reported no association with breast cancer. Similarly, for cervical and colon cancer development, some studies reported a significant association [41–46 and 49–56 respectively] whereas other studies reported no significant association [44, 47, 48, 57–61 respectively]. The same scenario persists for liver cancer: associated [62–68] and not “associated [69–71]; lung cancer: associated [72–77] and not associated [78–83]; prostate cancer: associated [11, 84–91] and not associated [92–98]; stomach cancer: claimed a significant association [106–112] and some other studies claimed insignificant association [111, 113–115]. Also, some GWA studies investigated the association of the IL-6 gene polymorphisms with thyroid cancer [116, 117], ovarian cancer [118], pancreatic cancer [119], neuroblastoma [120, 121] and renal cell carcinoma [122]. Thus we observed from the above discussion that different types of cancers were influenced by the three SNPs (rs1800795, rs1800796 and rs1800797) of the IL-6 gene. We also observed that the reported results varied across studies and therefore, remain inconclusive, which may be occurred due to the smaller sample size and different ethnic populations.

To overcome the ambiguity of GWAS findings, some Author’s performed meta-analysis based on only one of three important SNPs (rs1800795, rs1800796 and rs1800797) of the IL-6 gene or only one type of cancer to take more reliable and valid conclusion [123–129]. It should be mentioned here that a meta-analysis is conducted by the complete coverage of all relevant studies, solving the heterogeneity problem, and exploring the robustness of main findings using sensitivity analysis. Those meta-analysis reported that (i) the rs1800795 polymorphism of the IL-6 gene shows significant association with cervical [123] and colorectal [124] cancers, but insignificant association with stomach cancer [128, 129], (ii) thers1800796 polymorphism shows contradictory association with stomach cancer [111] and insignificant association with lung cancer [126, 127] and (iii) thers1800797 polymorphism shows insignificant association with colorectal cancer [124], stomach cancer [129] and all type of risks [128]. Thus those meta-analysis reports on the IL-6 gene were not consistent in their common issues. Zhou et al. [131] performed multi-case meta-analysis considering all of three important SNPs of the IL-6 gene as mentioned previously, three different ethnicities (Asian, African, Caucasian), nine types of cancers based on 49,408 cancers and 61,790 control cases. They reported that the IL-6 gene is significantly associated with the overall cancer risk. Particularly, they reported significant association of IL-6 gene with 2 types of cancer risks (liver and prostate) and insignificant association with 7 types of cancer risks (breast, cervical, colorectal, gastric, lung, lymphoma and myeloma) by the sub-group analysis of cancer types. Obviously, their specific report [130] contradicts with the results of other single-case meta-analyses [123–129] in the cases of their common interest, which may be happened due to smaller sample sizes, ethnicity and the deficiency of statistical modeling with the meta-dataset. For example, none of those meta-analyses [123–130] checked the model adequacy by the goodness of fit test. To estimate the combined effects, all of them used fixed effect (FE) or random effect (RE) models corresponding to the homogeneity or heterogeneity of effects which cannot give the guarantee of model adequacy [133].Therefore, in this paper, an attempt was made to provide a more comprehensive understanding about the association between the IL-6 gene SNPs (rs1800795, rs1800796, rs1800797) and different types of cancer risks, giving the weight on large sample size, more cancer types and appropriate statistical modeling based on the goodness of fit test [133] with the multi-case meta-dataset.

## Materials and methods

### Search strategy

Text mined data of the competent articles retrieved from PubMed, PubMed Central, Google Scholar, Web of Science and other online literature databases, published up to February 2019 in the English language were only considered for this meta-analysis. The following keywords were used for searching: (i) IL-6, (ii) IL-6, Cancer, (iii) IL-6, rs1800795, (iv) IL-6, rs1800796, (v) IL-6, rs1800797, (vi) IL-6 -174G/C or -572G/C or -597G/A, (vii) polymorphisms, (viii) GWAS, (ix) case-control study.

### Eligibility criteria

Two authors independently investigated the title and abstract for all papers and primarily removed the irrelevant and incomplete studies. For the final review the following inclusion-exclusion criteria were used: if the study was (i) designed to measures the association between the IL-6 gene polymorphisms (rs1800795, rs1800796, rs1800797) and cancer risk, (ii) case-control design and (iii) sufficient to provide necessary information of genotypic frequency, it selected for this meta-analysis.

### Data extraction

From the eligible studies several information was compiled for each selected study such as first author, year of the study, country of origin, ethnicity of the study subject, number of case-control, types of cancer, allelic and genotypic distribution and so on. To test the validity of any selected studies for this meta-analysis, Hardy-Weinberg equilibrium (HWE) test was performed using the Chi-square statistic. A selected study was considered as a good study for meta-analysis if Pr{χ2_obs_ ≤ χ2 } ≥ .05 (Table 1).

**Table 1:** Characteristic of eligible studies included in meta-analysis of the IL-6 gene (rs1800795, rs1800796, rs1800797) polymorphisms.

Study	Year	Country	Ethnicity	Cancer	Case/Control	HWE
rs1800795						
Zheng et al. [105]	2000	Sweden	Caucasian	Skin Cancer	73/128	0.357(Y)
El-Omar et al. [113]	2003	USA	Mixed	Stomach Cancer	213/209	0.913(Y)
Hwang (b) et al. [114]	2003	USA	Caucasian	Stomach Cancer	30/30	0.399(Y)
Hwang (a) et al. [114]	2003	USA	Asian	Stomach Cancer	30/30	1.000(Y)
Howell et al. [104]	2003	UK	Caucasian	Skin Cancer	161/224	0.258(Y)
Landi et al. [55]	2003	France	Caucasian	Colon Cancer	361/311	0.761(Y)
Sun et al. [98]	2004	USA	Caucasian	Prostate Cancer	1337/753	0.492(Y)
Bushley et al. [118]	2004	USA	Mixed	Ovarian Cancer	182/218	0.020(N)
Campa et al. [81]	2004	France	Caucasian	Lung Cancer	243/207	0.818(Y)
Smith et al. [34]	2004	UK	Caucasian	Breast Cancer	144/224	0.258(Y)
Zhang et al. [105]	2004	China	Caucasian	Skin Cancer	241/260	0.993(Y)
Campa et al. [79]	2005	France	Caucasian	Lung Cancer	1995/1982	0.448(Y)
Seifart et al. [80]	2005	Germany	Caucasian	Lung Cancer	182/243	0.163(Y)
Migita et al. [71]	2005	Japan	Asian	Liver Cancer	48/188	1.000(Y)
Hefler et al. [36]	2005	Austria	Caucasian	Breast Cancer	269/227	0.935(Y)
Snoussi et al. [33]	2005	Tunisia	African	Breast Cancer	305/200	0.829(Y)
Leibovici et al. [88]	2005	USA	Caucasian	Prostate Cancer	444/443	0.000(N)
Festa et al. [103]	2005	Sweden	Caucasian	Skin Cancer	241/260	0.993(Y)
Cordano et al. [23]	2005	UK	Caucasian	Blood Cancer	408/349	0.167(Y)
Basturk et al. [123]	2005	Turkey	Caucasian	Renal cell	25/49	0.007(N)
Mazur et al. [25]	2005	Poland	Caucasian	Blood Cancer	54/50	0.239(Y)
Kamangar et al. [115]	2006	Finland	Caucasian	Stomach Cancer	102/152	0.004(N)
Xing et al. [111]	2006	China	Asian	Stomach Cancer	65/71	0.141(Y)
Michaud et al. [97]	2006	USA	Caucasian	Prostate Cancer	484/613	0.832(Y)
Vairaktaris et al. [101]	2006	Greece	Caucasian	Oral Cancer	162/156	0.298(Y)
Cozen et al. [21]	2006	USA	Caucasian	Blood Cancer	146/125	0.333(Y)
Gunter et al. [61]	2006	USA	Caucasian	Colon Cancer	204/190	0.385(Y)
Theodoropoulos et al. [54]	2006	Greece	Caucasian	Colon Cancer	222/200	0.055(Y)
Noguetra et al. [45]	2006	Brazil	Mixed	Cervical Cancer	56/253	0.001(N)
Balasubramanian et al. [39]	2006	UK	Caucasian	Breast Cancer	497/490	0.759(Y)
Gonzalez-Zuloeta et al. [40]	2006	Netherland	Caucasian	Breast Cancer	171/3651	0.290(Y)
Lan et al. [22]	2006	USA	Caucasian	Blood Cancer	510/590	0.358(Y)
Rothman et al. [24]	2006	USA	Caucasian	Blood Cancer	3066/3499	0.506(Y)
Slattery et al. [62]	2007	USA	Caucasian	Colon Cancer	1579/1977	0.015(N)
Slattery et al. [27]	2007	USA	Caucasian	Breast Cancer	650/678	0.122(Y)
Deans et al. [107]	2007	UK	Caucasian	Stomach Cancer	197/224	0.258(Y)
Gatti et al. [108]	2007	Brazil	Mixed	Stomach Cancer	56/112	0.509(Y)
Duch et al. [19]	2007	Brazil	Mixed	Blood Cancer	52/60	0.442(Y)
Vishnoi et al. [70]	2007	India	Asian	Liver Cancer	124/200	0.936(Y)
Gonullu et al. [32]	2007	Turkey	Caucasian	Breast Cancer	38/24	0.000(N)
Vogel et al. [38]	2007	Denmark	Caucasian	Breast Cancer	361/361	0.728(Y)
Nearman et al. [20]	2007	USA	Caucasian	Blood Cancer	28/362	0.120(Y)
Crusius et al. [111]	2008	France	Caucasian	Stomach Cancer	243/1138	0.044(N)
Kesarwani et al. [96]	2008	India	Asian	Prostate Cancer	200/200	0.100(Y)
Vairaktaris et al. [100]	2008	Greece	Caucasian	Oral Cancer	162/156	0.000(N)
Colakogullari et al. [78]	2008	Turkey	Caucasian	Lung Cancer	44/58	0.221(Y)
Upadhyay et al. [67]	2008	India	Asian	Liver Cancer	168/201	0.586(Y)
Kury et al. [60]	2008	France	Caucasian	Colon Cancer	1023/1121	0.079(Y)
Wilkening et al. [53]	2008	Germany	Caucasian	Colon Cancer	303/580	0.481(Y)
Ennas et al. [18]	2008	Italy	Caucasian	Blood Cancer	39/112	0.506(Y)
Slattery et al. [51]	2009	USA	Caucasian	Colon Cancer	750/1250	0.016(N)
Slattery et al. [52]	2009	USA	Caucasian	Colon Cancer	1839/2014	0.015(N)
Gangwar et al. [41]	2009	India	Asian	Cervical Cancer	160/200	0.371(Y)
Ozgen et al. [117]	2009	Turkey	Caucasian	Thyroid Cancer	42/340	0.009(N)
Moore et al. [93]	2009	USA	Caucasian	Prostate Cancer	957/847	0.152(Y)
Pierce et al. [94]	2009	USA	Caucasian	Prostate Cancer	175/1934	0.132(Y)
Wang et al. [95]	2009	USA	Caucasian	Prostate Cancer	253/280	0.448(Y)
Zabaleta et al. [96]	2009	USA	Caucasian	Prostate Cancer	74/401	0.000(N)
Talar-Wojnarowska et al. [119]	2009	Poland	Caucasian	Pancreatic Cancer	97/50	0.191(Y)
Aladzsity et al. [16]	2009	Hungary	Caucasian	Blood Cancer	97/99	0.101(Y)
Falleti et al. [66]	2009	Italy	Caucasian	Liver Cancer	219/236	0.536(Y)
Ognjanovic et al. [69]	2009	USA	Mixed	Liver Cancer	117/221	0.000(N)
Tsilidis et al. [58]	2009	USA	Caucasian	Colon Cancer	203/367	0.537(Y)
Vasku et al. [59]	2009	Czech Republic	Caucasian	Colon Cancer	100/100	0.601(Y)
Cherel et al. [30]	2009	France	Caucasian	Breast Cancer	293/82	0.695(Y)
DeMichele et al. [31]	2009	USA	Caucasian	Breast Cancer	339/100	0.569(Y)
Andrie et al. [17]	2009	Greece	Caucasian	Blood Cancer	81/81	0.777(Y)
Ognjanovic et al. [50]	2010	USA	Mixed	Colon Cancer	271/539	0.000(N)
Zhao et al. [112]	2010	China	Asian	Stomach Cancer	142/200	0.943(Y)
Dossus et al. [87]	2010	Germany	Mixed	Prostate Cancer	7937/8508	0.035(N)
Cacev et al. [56]	2010	Croatia	Caucasian	Colon Cancer	160/160	0.582(Y)
Hawken et al. [63]	2010	Canada	Caucasian	Colon Cancer	1133/1125	0.461(Y)
Abuli et al. [49]	2011	Spain	Caucasian	Colon Cancer	1416/1424	0.672(Y)
Grimm et al. [46]	2011	Austria	Caucasian	Cervical Cancer	131/208	0.990(Y)
Gaur et al. [99]	2011	India	Caucasian	Oral Cancer	140/200	0.069(Y)
Giannitrapani et al. [65]	2011	Italy	Caucasian	Liver Cancer	105/95	0.402(Y)
Lima junior et al. [48]	2012	Brazil	Mixed	Cervical Cancer	345/345	0.093(Y)
Pooja et al. [28]	2012	India	Asian	Breast Cancer	200/200	0.000(N)
Totaro et al. [120]	2013	Italy	Caucasian	Neuroblastoma	326/511	0.646(Y)
Pohjnen et al. [109]	2013	Finland	Caucasian	Stomach Cancer	56/179	0.706(Y)
Chen et al. [72]	2013	China	Asian	Lung Cancer	1237/1252	0.903(Y)
Bai et al. [73]	2013	China	Asian	Lung Cancer	193/210	0.145(Y)
Oduor et al. [13]	2014	Kenya	African	Blood Cancer	117/88	1.000(Y)
Mandal et al. [85]	2014	USA	Caucasian	Prostate Cancer	164/140	0.001(N)
Gu et al. [14]	2014	China	Asian	Blood Cancer	157/435	0.159(Y)
Cao et al. [111]	2014	China	Asian	Stomach Cancer	162/162	0.210(Y)
Shi et al. [43]	2014	China	Asian	Cervical Cancer	518/518	0.349(Y)
Cil et al. [116]	2014	Turkey	Caucasian	Thyroid Cancer	190/216	0.722(Y)
Slattery et al. [29]	2014	USA	Mixed	Breast Cancer	3567/4157	0.000(N)
Chen et al. [89]	2015	China	Asian	Prostate Cancer	212/236	0.267(Y)
Talaat et al. [15]	2015	Egypt	Mixed	Blood Cancer	100/119	0.568(Y)
Sampaio et al. [110]	2015	Portugal	Caucasian	Stomach Cancer	50/50	0.608(Y)
Sa-Nguanraksa et al. [37]	2016	Thailand	Asian	Breast Cancer	391/79	0.000(N)
Pu et al. [42]	2016	China	Asian	Cervical Cancer	360/728	0.310(Y)
Abana et al. [26]	2017	USA	Caucasian	Breast Cancer	277/711	0.490(Y)
Winchester et al. [84]	2017	USA	Caucasian	Prostate Cancer	625/532	0.169(Y)
Attar et al. [106]	2017	Iran	Mixed	Stomach Cancer	100/361	0.000(N)
Sabrina et al. [44]	2017	Tunisia	African	Cervical Cancer	112/164	0.002(N)
DargahiAbbasabad et al. [86]	2018	Iran	Mixed	Prostate Cancer	112/250	0.000(N)
Zhao et al. [121]	2018	China	Asian	Neuroblastoma	130/50	0.585(Y)
Dos Santos et al. [112]	2018	Brazil	Mixed	Stomach Cancer	52/87	0.517(Y)
Taheri et al. [86]	2018	Iran	Mixed	Prostate Cancer	130/200	0.194(Y)
Shuwei Wang et al. [64]	2018	China	Asian	Colon Cancer	186/200	0.160(Y)
**rs1800796**						
Hwang (a) et al. [114]	2003	USA	Caucasian	Stomach Cancer	30/30	0.020(N)
Hwang (b) et al. [114]	2003	USA	Asian	Stomach Cancer	30/30	0.394(Y)
Sun et al. [98]	2004	USA	Caucasian	Prostate Cancer	1337/753	0.211(Y)
Xing et al. [111]	2006	China	Asian	Stomach Cancer	65/71	0.141(Y)
Seow et al. [75]	2006	Singapore	Asian	Lung Cancer	124/162	0.560(Y)
Kamanger et al. [116]	2006	USA	Caucasian	Stomach Cancer	102/152	0.004(N)
Slattery et al. [62]	2007	USA	Caucasian	Colon Cancer	1573/1972	0.015(N)
Bao et al. [91]	2008	China	Asian	Prostate Cancer	136/120	0.000(N)
Slattery et al. [51]	2009	USA	Caucasian	Colon Cancer	750/1205	0.000(N)
Kang et al. [111]	2009	Korea	Asian	Stomach Cancer	332/326	0.078(Y)
Pierce et al. [93]	2009	USA	Caucasian	Prostate Cancer	175/1934	0.161(Y)
Wang et al. [94]	2009	USA	Caucasian	Prostate Cancer	253/280	0.405(Y)
Tsilidis et al. [58]	2009	USA	Caucasian	Colon Cancer	203/367	0.019(N)
Su et al. [82]	2010	China	Asian	Lung Cancer	363/370	0.298(Y)
Lim et al. [83]	2011	Singapore	Asian	Lung Cancer	298/718	0.250(Y)
Bai et al. [73]	2013	China	Asian	Lung Cancer	193/210	0.145(Y)
Chen et al. [72]	2013	China	Asian	Lung Cancer	615/638	0.990(Y)
Liang et al. [74]	2013	China	Asian	Lung Cancer	138/138	0.625(Y)
Kiyohara et al. [77]	2014	Japan	Asian	Lung Cancer	462/379	0.919(Y)
Cao et al. [111]	2014	China	Asian	Stomach Cancer	162/162	0.210(Y)
Tang et al. [68]	2014	China	Asian	Liver Cancer	505/395	0.474(Y)
Chen et al. [89]	2015	China	Asian	Prostate Cancer	212/236	0.851(Y)
Haung et al. [90]	2016	China	Asian	Prostate Cancer	236/256	0.094(Y)
Zhang et al. [111]	2017	China	Asian	Stomach Cancer	473/474	0.750(Y)
Xie et al. [111]	2017	China	Asian	Stomach Cancer	400/400	0.859(Y)
Zhu et al. [35]	2017	China	Asian	Breast Cancer	1514/1540	0.204(Y)
Dos Santos et al. [112]	2018	Brazil	Mixed	Stomach Cancer	52/87	0.555(Y)
**rs1800797**						
Hwang et al. [114]	2003	USA	Caucasian	Stomach Cancer	30/30	0.399(Y)
Snoussi et al. [33]	2005	Tunisia	African	Breast Cancer	305/200	0.830(Y)
Festa et al. [102]	2005	Sweden	Caucasian	Skin Cancer	241/260	0.385(Y)
Rothman et al. [24]	2006	USA	Caucasian	Blood Cancer	2658/3068	0.124(Y)
Castro et al. [46]	2009	Sweden	Caucasian	Cervical Cancer	973/1763	0.584(Y)
Pierce et al. [93]	2009	USA	Caucasian	Prostate Cancer	175/1934	0.437(Y)
Tsilidis et al. [58]	2009	USA	Caucasian	Colon Cancer	203/362	0.931(Y)
Vasku et al. [89]	2009	Czech Republic	Caucasian	Colon Cancer	100/100	0.661(Y)
DeMichele et al. [31]	2009	USA	Caucasian	Breast Cancer	339/100	0.316(Y)
Gu et al. [14]	2014	China	Asian	Blood Cancer	93/204	0.831(Y)
Sa-Nguanraksa et al. [37]	2016	Thailand	Asian	Breast Cancer	391/79	0.863(Y)
Leng et al. [76]	2016	USA	Caucasian	Lung Cancer	242/336	0.346(Y)
Sabrina et al. [44]	2017	Tunisia	African	Cervical Cancer	112/164	0.000(N)
Winchester et al. [84]	2017	USA	Caucasian	Prostate Cancer	625/532	0.075(Y)
Huang et al. [57]	2018	USA	Caucasian	Colon Cancer	135/269	0.745(Y)
Dos Santos et al. [112]	2018	Brazil	Mixed	Stomach Cancer	52/87	0.446(Y)

HWE: Hardy-Weinberg equilibrium; Y: Yes; N: No; All the included studies are ordered by the year of publication.

### Statistical modeling for meta-analysis

Meta-analysis is a collection of statistical methods to compile the results of similar independent studies. It is used to take the overall decision across a number of similar studies. Let us now introduce the statistical methods that are used in this paper for taking the overall decision about the relationship between the IL-6 gene polymorphisms and cancer risk. At first we have checked the quality of existing studies by testing the Hardy-Weinberg equilibrium (HWE). The HWE test is performed using the Chi-square statistic with the null hypothesis that the genotypic ratio is consistent for the control population of all studies. The chi-square statistic for this test is given by:

$$\chi^{2}=\sum_{i=1}^{3}\frac{{\left(O_{i}-E_{i}\right)}^{2}}{E_{i}}$$ (1)

which follows chi-square distribution with 1 degree freedom. Here *O*_*i*_ and *E*_*i*_ represents observe and expected frequency of the genotype, respectively. If *p* and *q* are the probabilities of *C* and *G* allele, respectively and *O*_*i*_ = *obs(i)* is observe frequency of *i*th genotype among the 3 genotypes *CC*, *CG* and *GG*. Then *p* is calculated as:

$$p=\frac{2\times obs(CC)+obs(CG)}{2\times (obs(CC)+(obs(CG)+obs(GC))};\;\text{and}\;q=1-p$$ (2)

The expected frequency of *i*th genotype is denoted by *E*_*i*_ = *E(i)* defined as *E(CC) = p*^*2*^*n*, *E(CG) = 2pqn*, *E(GG) = q*^*2*^*n*, where *n* is the total number of observation.

The heterogeneity of different studies has been examined by using Cochran’s *Q* statistic and its extended Higgin’s & Thompson *I*^*2*^ statistic [131, 132]. The Cochran’s *Q* statistic is defined as:

$$Q={\sum_{k=1}^{K}w_{k}\left({\hat{\theta }}_{k}-\frac{\sum_{k=1}^{K}w_{k}{\hat{\theta }}_{k}}{\sum_{k=1}^{K}w_{k}}\right)}^{2},$$ (3)

which follows the chi-square distribution with *K*-1 degrees of freedom. Here ${\hat{\theta }}_{k}=\ln\left({OR}_{k}\right)$ for the *k*th study, and $w_{k}=\frac{1}{{\hat{\sigma }}_{k}^{2}}$ is the weight of *k*th study. The variance of the *k*th study can be calculated as:

$${\hat{\sigma }}_{k}^{2}=var\;(\text{ln}\;(OR_{k}))=\frac{1}{m_{1k}}+\frac{1}{m_{2k}}+\frac{1}{m_{3k}}+\frac{1}{m_{4k}}$$ (4)

where *m*_1*k*_ and *m*_2*k*_ indicates the number of exposures and *m*_3*k*_ and *m*_4*k*_ indicates non-exposures, in the case-control groups of *k*th study, respectively (that is, for the genetic model *C* vs. *G*, the allele *C* is exposer and *G* is non-exposer). The Higgin’s& Thompson *I*^*2*^ statistic is defined as:

$$I^{2}=\max\{0,\frac{Q-(K-1)}{Q}\;\times 100\%\}$$ (5)

The values of *I*^*2*^ greater than 25%, 50% and 75% indicates the low, moderate, and high heterogeneity among the individual studies, respectively.

The pooled odds ratio (OR) has been applied for checking the significant association between the IL-6 gene polymorphisms and cancer risk under different genetic models like as dominant models [CC + CG vs. GG or AA + AG vs. GG], homozygote models [CC vs. GG or AA vs. GG], over-dominant models [CG vs. CC + GG or AG vs. AA + GG], recessive models [CC vs. CG + GG or AA vs. AG + GG], and allelic contrast models [C vs. G or A vs. G]. To calculate pooled OR for each genetic combination, we have used the random effect model if the *Q*-test suggests the highly significant heterogeneity (*p*-value < 0.10) among different studies; otherwise, fixed effect model are used. We have also estimated 95% confidence interval (CI) of OR based on *Z*-statistic [131, 132]. The OR for the *k*th study is calculated as:

$$OR_{k}=\frac{\frac{m_{1k}}{m_{2k}}}{\frac{m_{3k}}{m_{4k}}}=\frac{m_{1k}m_{4k}}{m_{2k}m_{3k}},$$ (6)

For the fixed effect model, overall OR is calculated by using the Mentel—Haenszel (M-H) method as follows:

$${\hat{\theta }}_{k}=\theta_{F}+\epsilon_{k};\;\text{where}\;\epsilon_{k}∼N(0,{\hat{\sigma }}_{k}^{2})$$ (7)

where ${\hat{\theta }}_{F}={\hat{OR}}_{MH}=\frac{\sum_{k=1}^{K}\;\left(\frac{m_{1k}m_{4k}}{N_{k}}\right)\;}{\sum_{k=1}^{K}\;\left(\frac{m_{2k}m_{3k}}{N_{k}}\right)}$

$$=\sum_{k=1}^{K}\left(\frac{\frac{m_{2k}m_{3k}}{N_{k}}}{\sum_{i=1}^{K}\left(\frac{m_{2k}m_{3k}}{N_{k}}\right)}\right)\times OR_{k},$$ (8)

and *N*_*k*_ *= m*_1*k*_*+ m*_2*k*_ *+ m*_3*k*_ *+ m*_4*k*_, and the variance and 95% C.I. of overall effect can be defined as:

$$Var\;({\hat{\theta }}_{F})\frac{1}{\sum_{k=1}^{K}\left(\frac{m_{2k}m_{3k}}{N_{k}}\right)};{\hat{\theta }}_{F}\pm 1.96\sqrt{Var\;({\hat{\theta }}_{F})},$$ (9)

For the random effect model, overall OR is calculated by using the inverse variance method as follows:

$${\hat{\theta }}_{k}=\theta_{R}+v_{k}+\epsilon_{k};\;\text{where},v_{k}∼N(0,\tau^{2})$$ (10)

The random parameter *θ*_*R*_ is calculated as,

$${\hat{\theta }}_{R}=\frac{\sum_{k=1}^{K}w_{kR}{\hat{\theta }}_{k}}{\sum_{k=1}^{K}w_{kR}},$$ (11)

Where

$$se({\hat{\theta }}_{R})=\sqrt{var({\hat{\theta }}_{R})}=\sqrt{\frac{1}{\sum_{k=1}^{K}w_{kR}}};$$

$$w_{kR}=\frac{1}{\sigma_{k}^{2}+\tau^{2}},\;\text{and}\;\tau^{2}=\frac{Q-(K-1)}{\sum w_{k}-\left(\frac{\sum w_{k}^{2}}{\sum w_{k}}\right)}$$ (12)

However, *Q*-test cannot give the assurance of model adequacy. Therefore we also considered the goodness of fit test to check the model adequacy. To check the model adequacy, we performed three distinct goodness of fit (GoF) tests proposed by Chen et al. [133]. These three GoF tests known as Anderson-Darling (AD) test [134, 135], Cramer-von Mises (CvM) test [135–137] and Shapiro-Wilk (SW) test [138] for testing the null hypothesis that the individual effects follow the normal distribution. If individual effects are significantly normal, then random effect model is used for estimating the combined effect else fixed effect model is used. The test statistic of each normality test is defined as:

$$AD=-K-\sum_{k=1}^{K}\left(\frac{2k-1}{k}\right)\left[\ln F({\hat{\theta }}_{k})+\ln(1-F({\hat{\theta }}_{K+1-k}))\right],$$ (13)

$$CvM=\frac{1}{12K}+{\sum_{k=1}^{K}\left[\frac{2k-1}{2K}-F({\hat{\theta }}_{k})\right]}^{2},$$ (14)

$$SW=\frac{{\left(\sum_{k=1}^{K}a_{k}{\hat{\theta }}_{k}\right)}^{2}}{\sum_{k=1}^{K}{\left({\hat{\theta }}_{k}-\bar{\theta }\right)}^{2}},$$ (15)

where, ${\hat{\theta }}_{k}$ is the ordered data, $\bar{\theta }$ is sample mean of ${\hat{\theta }}_{k}$, *K* is sample size means number of individual study, *F*(${\hat{\theta }}_{k}$) is cumulative distribution function of normal distribution with *k*th order statistic, *a*_*k*_ is constants generated from means, variances, and covariances of the order statistics. To perform these three tests, Chen et al. [133] proposed the following steps:

**Step 1.**Compute ad_0_, cvm_0_, and sw_0_ from AD, CvM, and SW statistics, respectively, for given ${\hat{\theta }}_{k}$ = ln(OR_*k*_), *k* = 1, 2, …, *K*;

**Step 2.** Resample B = 10^5^ sub-samples from $MVN(0,\hat{\Sigma }$), where,

$$\hat{\sum }=\left[\begin{array}{llll} {\hat{\sigma }}_{1}^{2}+{\hat{\tau }}^{2} & 0 & \cdots & 0\\ 0 & {\hat{\sigma }}_{2}^{2}+{\hat{\tau }}^{2} & \cdots & 0\\ \vdots & \vdots & \ddots & \vdots \\ 0 & 0 & \cdots & {\hat{\sigma }}_{K}^{2}+{\hat{\tau }}^{2} \end{array}\right]$$

Then, compute ad_*j*_, cvm_*j*_, and sw_*j*_ by using AD, CvM, and SW statistics, respectively, for each sample *j* (*j* = 1, 2, …,*B)*.

**Step 3.** Compute *p-*values by using $\sum_{j=1}^{B}I_{\left[{ad}_{j}\;>{ad}_{0}\right]}/B,\;\sum_{j=1}^{B}I_{\left[{cvm}_{j}\;>{cvm}_{0}\right]}/B$ and $\sum_{j=1}^{B}I_{\left[{sw}_{j}<{sw}_{0}\right]}/B$ for the above three tests, respectively, where Is>s0=1fors>s0;0,otherewise.

Then the respective *z*-score is calculated as follows:

$$Z=\{\begin{array}{l} \frac{\sum_{k}w_{k}{\hat{\theta }}_{k}}{\sqrt{\sum_{k}w_{k}}},\;\;\text{for}\;\text{fixed}\;\text{effect}\;\text{model}\\ \frac{\sum_{k}w_{kR}{\hat{\theta }}_{k}}{\sqrt{\sum_{k}w_{kR}}},\;\text{for}\;\text{random}\;\text{effect}\;\text{model} \end{array}$$ (16)

Subgroup analyses are also executed based on ethnicity and type of cancer by using the techniques mentioned above.

We have performed the sensitivity analysis using the full data and the reduced data that are obtained by removing the studies those are failed to pass the HWE validation and publication bias test. The publication bias is examined for each study visually by funnel plot and significantly by Egger regression test [139] and Begg’s test [140]. The Egger regression test statistic is defined as:

$$T=\frac{\hat{a}}{se(\hat{a})}$$ (17)

which follows the *t-*distribution with (*K*-2) degrees of freedom under the null hypothesis H_0_: *α* = 0 (no publication bias), $\hat{\alpha }$ is obtained by the least square estimation using one of the following models:

$${\hat{\theta }}_{k}\sqrt{w_{k}}=\alpha +\mu \sqrt{w_{k}}+\varepsilon_{k},\;\text{for}\;\text{fixed}\;\text{effect}\;\text{model},\;\text{and}$$ (18)

$${\hat{\theta }}_{k}\sqrt{w_{kR}}=\alpha +\mu \sqrt{w_{kR}}+\varepsilon_{k},\;\text{for}\;\text{random}\;\text{effect}\;\text{model},$$ (19)

where *ε*_*k*_ ~ *iid N*(0, *σ*^2^). The Begg’s test statistic is defined as:

$$Z=\frac{C-D}{\sqrt{K(K-1)(2K+5)/18}},$$ (20)

which follows asymptotically *N*(0,1) under the null hypothesis H_0_: *α* = 0 (no publication bias). Here *C* and *D* are the number of concordant and discordant, respectively, those are obtained by using the Kendall’s ranking of $t_{k}^{*}$ and ${\hat{\sigma }}_{k}^{2}$or ${\hat{\sigma }}_{kR}^{2}$. Here:

$$t_{k}^{*}=\frac{t_{k}-\bar{t}}{\sqrt{\vartheta_{k}^{*}}}$$ (21)

where, *t*_*k*_ = OR_*k*_ is the OR of *k*th study, and:

$$\bar{t}=\{\begin{array}{l} \frac{\sum_{k}w_{k}t_{k}}{\sqrt{\sum_{k}w_{k}}},\;\;\text{for}\;\text{fixed}\;\text{effect}\;\text{model}\\ \frac{\sum_{k}w_{kR}t_{k}}{\sqrt{\sum_{k}w_{kR}}},\;\text{for}\;\text{random}\;\text{effect}\;\text{model} \end{array}$$ (22)

$$\vartheta_{k}^{*}=\{\begin{array}{l} {\hat{\sigma }}_{k}^{2}-\frac{1}{\sum w_{k}},\;\;\text{for}\;\text{fixed}\;\text{effect}\;\text{model}\\ {\hat{\sigma }}_{kR}^{2}-\frac{1}{\sum w_{kR}},\;\text{for}\;\text{random}\;\text{effect}\;\text{model} \end{array}$$ (23)

We have used the ‘meta’ R-package (http://meta-analysis-with-r.org/) for implementing the above statistical methods for the meta-analysis.

## Results

### Study characteristics

**Fig 1:**
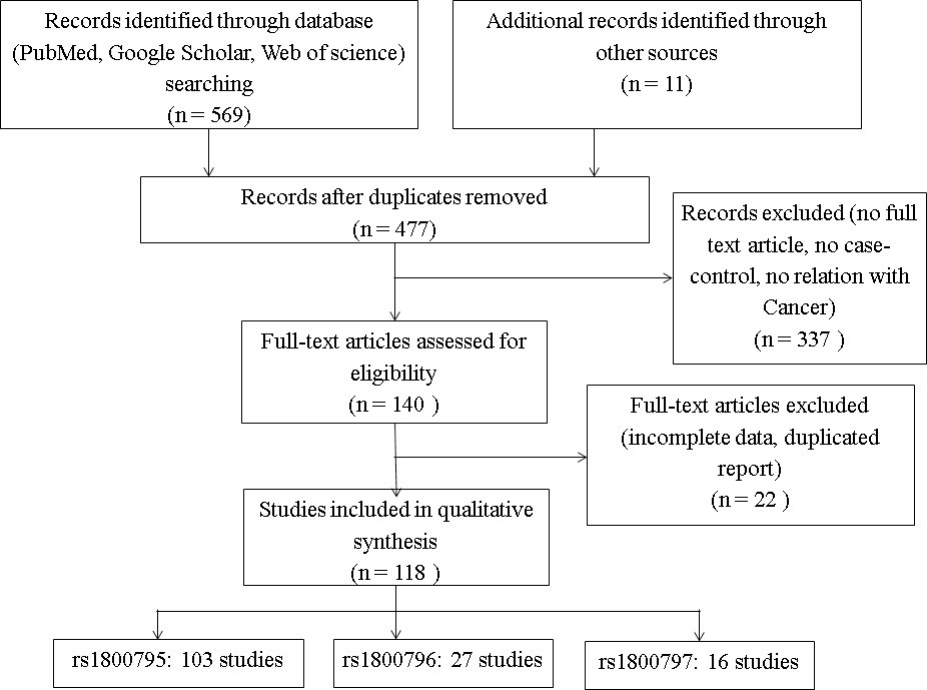
Flow diagram of study selection for the IL-6 gene polymorphisms; where ‘n’ is the number of studies.

In this meta-analysis, first we reviewed 580 articles which mentioned the IL-6 gene in their titles and abstracts. Then 477 articles were selected after removing the duplication. Again we removed 337 articles due to the absence of full text, case-control and cancer related studies. Finally 118 articles were selected for final review by removing some studies having incomplete information. The flow chart of the studies selection process was shown in Fig 1. The finally selected articles included 103 studies for the rs1800795 SNP with 45238 cases and 57255 controls (Table 2), 27 studies for the rs1800796 SNP with 10733 cases and 13405 controls (Table 3), 16 studies for the rs1800797 SNP with 6674 cases and 9493 controls (Table 4). These articles were classified to different types of cancer such as blood cancer, breast cancer, cervical cancer, colon cancer, liver cancer, lung cancer, neuroblastoma, oral cancer, ovarian cancer, pancreatic cancer, prostate cancer, skin cancer, stomach cancer and thyroid cancer. For being the single study, ovarian cancer, renal cell carcinoma (RCC) and pancreatic cancer for the rs1800795 SNP, breast cancer and liver cancer for rs1800796 SNP and, lung cancer and skin cancer for the rs1800797 SNP were organized in a subgroup entitled as other cancer (Tables 2–4).

**Table 2:** Meta-analysis of the IL-6 -174G/C polymorphism association with cancer risk.

			CC vs. GG		CC vs. CG + GG		CC + CG vs. GG		CG vs. CC + GG		C vs. G	
	Study Number	Sample Size	OR^a^ (95% CI^b^)	*p*-value	OR (95% CI)	*p*-value	OR (95% CI)	*p*-value	OR (95% CI)	*p*-value	OR (95% CI)	*p*-value
**Overall**	103	102493	1.06 [0.98; 1.16]	0.1429	1.05 [0.98; 1.12]	0.2054	1.02 [0.99; 1.05]	0.0615	0.99 [0.96; 1.01]	0.2681	1.02 [0.97; 1.06]	0.4459
**Blood Cancer**	13	10824	1.04 [0.86; 1.26]	0.6246	1.06 [0.86; 1.29]	0.6289	0.98 [0.87; 1.10]	0.7399	0.99 [0.90; 1.08]	0.7508	1.01 [0.96; 1.07]	0.8411
**Breast Cancer**	14	18686	0.93 [0.84; 1.02]	0.1329	0.96 [0.88; 1.05]	0.4042	0.95 [0.82; 1.11]	0.5291	0.98 [0.91; 1.04]	0.4804	1.00 [0.90; 1.11]	0.9619
**Cervical Cancer**	7	4098	**1.63 [1.06; 2.52]**	**0.0266**	**1.57 [1.25; 1.97]**	**0.0001**	**1.31 [1.05; 1.64]**	**0.0178**	1.14 [0.89; 1.47]	0.2927	**1.29 [1.07; 1.56]**	**0.0075**
**Colon Cancer**	15	21263	0.98 [0.85; 1.13]	0.8097	0.98 [0.87; 1.08]	0.4136	1.02 [0.92; 1.14]	0.6893	1.02 [0.97; 1.08]	0.4196	1.01 [0.93; 1.09]	0.7632
**Liver Cancer**	6	1922	**0.61 [0.42; 0.88]**	**0.0082**	**0.68 [0.48; 0.97]**	**0.0321**	**0.69 [0.56; 0.85]**	**0.0004**	0.75 [0.54; 1.05]	0.0908	**0.73 [0.62; 0.86]**	**0.0002**
**Lung Cancer**	6	7846	1.08 [0.85; 1.36]	0.5296	0.97 [0.76; 1.23]	0.7981	1.08 [0.86; 1.84]	0.5137	1.07 [0.86; 1.34]	0.5334	1.01 [0.87; 1.18]	0.9102
**Neuroblastoma**	2	1017	1.18 [0.71; 1.95]	0.5244	1.15 [0.71; 1.88]	0.5731	1.06 [0.84; 1.81]	0.6865	1.05 [0.80; 1.37]	0.7259	1.08 [0.88; 1.33]	0.4570
**Oral Cancer**	3	896	0.69 [0.08; 6.27]	0.7430	0.74 [0.18; 3.04]	0.6714	0.80 [0.16; 4.09]	0.7864	0.86 [0.25; 2.95]	0.8160	0.81 [0.25; 2.58]	0.7197
**Prostate Cancer**	14	28441	**1.24 [1.05; 1.46]**	**0.0096**	**1.19 [1.03; 1.39]**	**0.0202**	1.09 [0.98; 1.22]	0.1142	0.97 [0.91; 1.05]	0.4773	**1.09 [1.00; 1.19]**	**0.0441**
**Skin Cancer**	4	1588	0.99 [0.75; 1.32]	0.968	0.96 [0.76; 1.20]	0.6564	1.05 [0.84; 1.31]	0.6808	1.08 [0.88; 1.31]	0.4597	1.00 [0.87; 1.16]	0.9912
**Stomach Cancer**	14	4503	1.12 [0.85; 1.50]	0.4087	1.07 [0.81; 1.40]	0.6405	1.08 [0.92; 1.22]	0.4317	1.04 [0.90; 1.19]	0.6024	1.04 [0.94; 1.16]	0.4517
**Thyroid Cancer**	2	788	1.20 [0.67; 2.13]	0.5364	1.30 [0.75; 2.27]	0.3477	0.87 [0.63; 1.21]	0.4149	0.79 [0.57; 1.10]	0.1680	0.97 [0.76; 1.25]	0.8399
**Other Cancers**	3	621	1.10 [0.58; 2.10]	0.7683	1.09 [0.73; 1.61]	0.6802	1.29 [0.80; 2.13]	0.3236	1.07 [0.74; 1.56]	0.7148	1.14 [0.85; 1.54]	0.3711
**Ethnicity**^**C**^												
**African**	3	986	1.58 [0.68; 3.66]	0.2895	1.40 [0.61; 3.25]	0.4248	**1.66 [1.20; 2.3]**	**0.0027**	**1.64 [1.17; 2.30]**	**0.0037**	**1.54 [1.16; 2.04]**	**0.0030**
**Asian**	19	10043	**1.56 [1.19; 2.03]**	**0.0011**	**1.37 [1.22; 1.53]**	**0.0001**	**1.17 [1.08; 1.29]**	**0.0024**	0.93 [0.85; 1.02]	0.1151	**1.20 [1.12; 1.29]**	**0.00001**
**Caucasian**	66	62535	1.01[0.92; 1.11]	0.8193	1.00 [0.94; 1.07]	0.9469	1.00 [0.97; 1.04]	0.8788	1.00 [0.97; 1.04]	0.9164	1.00 [0.95; 1.05]	0.9291
**Mixed**	15	28929	0.97 [0.74; 1.27]	0.8224	1.02 [0.80; 1.31]	0.8629	0.89 [0.77; 1.04]	0.1493	0.89 [0.78; 1.03]	0.1134	0.93 [0.82; 1.06]	0.2676

Statistical significance presented in Bold.

^a^Odds Ratio;

^b^Confidence Interval ORs for the ethnicity subgroups are for overall cancer risk.

**Table 3:** Meta-analysis of the IL-6 -572G/C polymorphism association with cancer risk.

			CC vs. GG		CC vs. CG + GG		CC + CG vs. GG		CG vs. CC + GG		C vs. G	
	Study Number	Sample size	OR^a^ (95% CI^b^)	*p*-value	OR (95% CI)	*p*-value	OR (95% CI)	*p*-value	OR (95% CI)	*p*-value	OR (95% CI)	*p*-value
**Overall**	27	24138	1.03 [0.85; 1.25]	0.7635	0.99 [0.86; 1.14]	0.8582	1.07 [0.94; 1.22]	0.2931	**1.12 [1.01; 1.23]**	**0.0288**	1.04 [0.95; 1.15]	0.3839
**Colon Cancer**	3	6070	1.04 [0.67; 1.64]	0.8507	1.05 [0.73; 1.50]	0.8000	1.04 [0.67; 1.63]	0.8507	1.07 [0.85; 1.36]	0.5552	1.10 [0.79; 1.53]	0.5613
**Lung Cancer**	7	4808	1.13 [0.75; 1.69]	0.5575	0.93 [0.74; 1.17]	0.5400	**1.31 [1.04; 1.65]**	**0.0228**	**1.31 [1.08; 1.59]**	**0.0072**	1.19 [0.98; 1.43]	0.0734
**Prostate Cancer**	6	5928	**0.52 [0.37; 0.72]**	**0.0001**	**0.67 [0.53; 0.84]**	**0.0005**	**0.74 [0.61; 0.90]**	**0.0025**	1.00 [0.84; 1.18]	0.9811	**0.74 [0.64; 0.85]**	**0.0000**
**Stomach Cancer**	9	3378	**1.41 [1.10; 1.81]**	**0.0076**	**1.29 [1.07; 1.55]**	**0.0080**	**1.41 [1.09; 1.81]**	**0.0088**	1.02 [0.79; 1.31]	0.8940	**1.16 [1.03; 1.30]**	**0.0069**
**Other Cancer**	2	3954	1.13 [0.47; 2.68]	0.7852	0.90 [0.78; 1.04]	0.1400	1.03 [0.64; 1.67]	0.8922	1.12 [0.99; 1.28]	0.0841	1.05 [0.72; 1.53]	0.7878
**Ethnicity**^C^												
**Asian**	18	12883	1.02 [0.79; 1.31]	0.8812	1.00 [0.83; 1.20]	0.9636	1.06 [0.91; 1.25]	0.4557	**1.13 [1.01; 1.27]**	**0.0293**	1.04 [0.92; 1.19]	0.4974
**Caucasian**	8	11116	1.07 [0.76; 1.49]	0.7105	0.97 [0.78; 1.21]	0.7964	1.10 [0.87; 1.39]	0.4398	1.12 [0.89; 1.40]	0.3391	1.04 [0.87; 1.26]	0.6445
**Mixed**	1	139	3.20 [.28; 36.45]	0.3487	3.44 [.30; 38.90]	0.3181	0.83 [0.37; 1.86]	0.6590	0.70 [0.30; 1.63]	0.4130	0.97 [0.48; 1.98]	0.9379

Statistical significance presented in Bold.

^a^Odds Ratio;

^b^Confidence Interval;

^C^ ORs for the ethnicity subgroups are for overall cancer risk.

**Table 4:** Meta-analysis of the IL-6 -597G/A polymorphism association with cancer risk.

			AA vs. GG		AA vs. AG + GG		AA + AG vs. GG		AG vs. AA + GG		A vs. G	
	Study Number	Sample size	OR^a^ (95% CI^b^)	*p*-value	OR (95% CI)	*p*-value	OR (95% CI)	*p*-value	OR (95% CI)	*p*-value	OR (95% CI)	*p*-value
**Overall**	16	16167	0.96 [0.85; 1.08]	0.5152	0.97 [0.87; 1.07]	0.5064	1.00 [0.93; 1.08]	0.9289	0.98 [0.91; 1.05]	0.5025	0.99 [0.94; 1.04]	0.7169
**Blood Cancer**	2	6023	0.97 [0.83; 1.13]	0.7353	0.90 [0.84; 1.12]	0.6699	1.01 [0.47; 6.86]	0.8045	0.97 [0.88; 1.07]	0.5815	1.75 [0.48; 6.46]	0.3981
**Breast Cancer**	3	1414	1.11 [0.63; 1.92]	0.7097	1.07 [0.64; 1.79]	0.7980	1.24 [0.82; 1.88]	0.0800	0.79 [0.59; 1.06]	0.1176	1.20 [0.95; 1.52]	0.1221
**Cervical Cancer**	2	3012	**0.79 [0.63; 0.98]**	**0.0390**	**0.82 [0.68; 1.00]**	**0.0474**	0.93 [0.75; 1.23]	0.3969	0.94 [0.80; 1.09]	0.4312	0.91 [0.82; 1.02]	0.0667
**Colon Cancer**	3	1174	0.84 [0.60; 1.20]	0.3674	0.86 [0.64; 1.18]	0.3700	0.93 [0.72; 1.20]	0.5885	0.97 [0.77; 1.24]	0.8488	0.93 [0.78; 1.10]	0.3887
**Prostate Cancer**	2	3266	0.97 [0.71; 1.31]	0.8243	1.07 [0.81; 1.42]	0.6183	0.87 [0.72; 1.05]	0.1708	1.17 [0.97; 1.41]	0.0944	0.95 [0.83; 1.09]	0.4591
**Stomach Cancer**	2	199	1.71 [0.54; 5.34]	0.3551	1.83 [0.60; 5.57]	0.2846	0.98 [0.54; 1.76]	0.9657	1.21 [0.67; 2.20]	0.5301	1.10 [0.69; 1.76]	0.6853
**Other Cancers**	2	1079	1.36 [0.96; 1.92]	0.0744	1.20 [0.90; 1.60]	0.2122	1.29 [0.96; 1.73]	0.0524	0.91 [0.71; 1.16]	0.4556	**1.19 [1.00; 1.41]**	**0.0450**
**Ethnicity**^C^												
**African**	2	781	0.80 [0.37; 1.70]	0.5600	0.69 [0.32; 1.46]	0.3323	**1.48 [1.08; 2.03]**	**0.0135**	**0.61 [0.44; 0.84]**	**0.001**	**1.28 [0.98; 1.67]**	**0.0191**
**Asian**	2	767	0.61 [.02; 15.10]	0.7600	0.62 [0.02; 15.24]	0.7669	2.11 [0.91; 4.85]	0.0602	0.56 [0.11; 2.89]	0.0800	2.11 [0.93; 4.81]	0.0753
**Caucasian**	11	14480	0.97 [0.85; 1.10]	0.5965	0.97 [0.87; 1.08]	0.5689	0.98 [0.91; 1.05]	0.5254	1.00 [0.93; 1.07]	0.9640	0.97 [.87; 1.08]	0.5689
**Mixed**	1	139	1.29 [0.36; 4.67]	0.6900	1.44 [0.42; 4.96]	0.5672	0.86 [0.43; 1.72]	0.6774	1.30 [0.65; 2.62]	0.4600	0.98 [0.57; 1.04]	0.9343

Statistical significance presented in Bold.

^a^Odds Ratio;

^b^Confidence Interval;

^C^ ORs for the ethnicity subgroups are for overall cancer risk.

### Quantitative synthesis

#### IL-6 rs1800795 SNP

In the overall analysis, we found that the rs1800795 SNP was not associated with overall risk of cancer under five genetic models [C vs. G: OR = 1.02, 95% CI = 0.97–1.06, p-value = 0.445; CC vs. GG: OR = 1.06, 95% CI = 0.98–1.16, p-value = 0.1429; CC vs. CG + GG: OR = 1.05, 95% CI = 0.98–1.12, p-value = 0.2054; CC + CG vs. GG: OR = 1.02, 95% CI = 0.99–1.05, p-value = 0.0615; CG vs. CC + GG: OR = 0.99, 95% CI = 0.96–1.01, p-value = 0.2689] (Table 2 and S1A–S1E Fig in S1 File).

The subgroup analysis through the types of cancer showed the significant association that the IL-6 -174G/C polymorphism performed a protective role in liver cancer for four genetic models [C vs. G: OR = 0.73, 95% CI = 0.62–0.86, p-value = 0.0002; CC vs. GG: OR = 0.61, 95% CI = 0.42–0.88, p-value = 0.0082; CC vs. CG + GG: OR = 0.68, 95% CI = 0.48–0.97, p-value = 0.0.0321; CC + CG vs. GG: OR = 0.69, 95% CI = 0.56–0.85, p-value = 0.0004]; increased the risk for cervical cancer under four genetic models [C vs. G: OR = 1.29, 95% CI = 1.07–1.56, p-value = 0.0075; CC vs. GG: OR = 1.63, 95% CI = 1.06–2.52, p-value = 0.0266; CC vs. CG + GG: OR = 1.57, 95% CI = 1.25–1.97, p-value = 0.0001; CC + CG vs. GG: OR = 1.31, 95% CI = 1.05–1.64, p-value = 0.0178]; as well as increasing the risk for prostate cancer [C vs. G: OR = 1.09, 95% CI = 1.00–1.19, p-value = 0.0441; CC vs. GG: OR = 1.24, 95% CI = 1.05–1.46, p-value = 0.0096; CC vs. CG + GG: OR = 1.19, 95% CI = 1.03–1.39, p-value = 0.0202]. The blood cancer, breast cancer, colon cancer, lung cancer, neuroblastoma, oral cancer, skin cancer, stomach cancer, thyroid cancer, ovarian cancer and pancreatic cancer showed insignificant associations with the IL-6 -174G/C polymorphism (Table 2).

The subgroup analysis according to ethnicity showed that the IL-6 -174G/C polymorphism was not significantly associated with the cancer risk of Caucasian and mixed populations (Table 2). The subgroup analysis showed the significant association with the increasing overall cancer risk of Asian population under four genetic models [C vs. G: OR = 1.20, 95% CI = 1.12–1.29, p-value = 0.0000; CC vs. GG: OR = 1.56, 95% CI = 1.08–2.03, p-value = 0.0011; CC vs. CG + GG: OR = 1.37, 95% CI = 1.22–1.53, p-value = 0.0001; CC + CG vs. GG: OR = 1.17, 95% CI = 1.08–1.29, p-value = 0.0024] and African population [C vs. G: OR = 1.54, 95% CI = 1.16–2.04, p-value = 0.0030; CC + CG vs. GG: OR = 1.66, 95% CI = 1.20–2.30, p-value = 0.0027; CG vs. CC + GG: OR = 1.64, 95% CI = 1.17–2.30, p-value = 0.0037] (Table 2).

#### Source of heterogeneity

We observed significant heterogeneity in the analysis of the IL-6 rs1800795 (-174G /C) polymorphism for overall cancer [CC vs.GG: Q = 258.44, df = 97, p-value = 0.0001,τ^2^ = 0.0747, I^2^ = 62.47%; CC vs. CG + GG: Q = 228.48, df = 97, p-value = 97, τ^2^ = .0478, I^2^ = 57.53%; CC + CG vs. GG: Q = 333.19, df = 100, p-value = 0.0001, τ^2^ = .0485, I^2^ = 69.98%; CG vs. CC + GG: Q = 297.07, df = 100, p-value = 0.0001, τ^2^ = .0385, I^2^ = 66.34%; C vs. G: Q = 378.15, df = 100, p-value = 0.0001, τ^2^ = .0290, I^2^ = 73.56%]. The subgroup analysis corresponding to cancer type and ethnicity were performed to observe the sources of heterogeneity. The results of our analysis suggested that the studies in breast cancer, cervical cancer, colon cancer, lung cancer, oral cancer, prostate cancer, stomach cancer, and the ethnicity of Asian, Caucasian and Mixed population were the main sources of heterogeneity (S1 Table).

#### IL-6 rs1800796 SNP

The results generated through this meta-analysis showed that the IL-6 -572G/C polymorphism was significantly associated with the overall cancer risk in the case of over-dominant model [CG vs. CC + GG: OR = 1.12, 95% CI = 1.01–1.23, p-value = 0.0288] (Table 3 and S1F–S1J Fig in S1 File). Though, it was not significantly associated with the overall cancer risk under the other four genetic models (Allelic, dominant, recessive and homozygote).

The subgroup analysis through the types of cancer showed the significant association that the IL-6 rs1800796 (-572G/C) performed a protective role in prostate cancer for four genetic models [C vs. G: OR = 0.74, 95% CI = 0.64–0.85, p-value = 0.0000; CC vs. GG: OR = 0.52, 95% CI = 0.37–0.72, p-value = 0.0001; CC vs. CG + GG: OR = 0.67, 95% CI = 0.53–0.84, p-value = 0.0005; CC + CG vs. GG: OR = 0.74, 95% CI = 0.61–0.90, p-value = 0.0025]. The IL-6 -572G/C polymorphism was also exhibited significant association with the increasing risk of stomach cancer under four genetic models [C vs. G: OR = 1.16, 95% CI = 1.03–1.30, p-value = 0.0069; CC vs. GG: OR = 1.41, 95% CI = 1.10–1.81, p-value = 0.0076; CC vs. CG + GG: OR = 1.29, 95% CI = 1.07–1.55, p-value = 0.0080; CC + CG vs. GG: OR = 1.41, 95% CI = 1.09–0.81, p-value = 0.0088] and lung cancer for genetic models [CC + CG vs. GG: OR = 1.31, 95% CI = 1.04–1.65, p-value = 0.0228; CG vs. CC + GG: OR = 1.31, 95% CI = 1.08–1.59, p-value = 0.0072]. The colon, breast and liver cancers showed insignificant association with this polymorphism (Table 3).

The subgroup analysis based on ethnicity, the Asian population suggested that the IL-6 -572G/C polymorphism was significantly associated with increasing overall cancer risk for the over-dominant model [CG vs. CC + GG: OR = 1.13, 95% CI = 1.01–1.27, p-value = 0.0293]. The Caucasian and mixed ethnic group showed insignificant association of the IL-6 -572G/C polymorphism with the overall cancer risk (Table 3).

#### Source of heterogeneity

We found the significant heterogeneity of different studies in the analysis of IL-6 -572G/C polymorphism for overall cancer risk under the all genetic models [C vs. G: Q = 89.96, df = 26, p-value = 0.0001, τ^2^ = .0391, I^2^ = 71.04; CC vs. GG: Q = 55.82, df = 26, p-value = 0.0006, τ^2^ = .1033, I^2^ = 53.41%; CC vs. CG + GG: Q = 49.23, df = 26, p-value = .0039, τ^2^ = .0459, I^2^ = 47.18%; CC + CG vs. GG: Q = 76.19, df = 26, p-value = .0001, τ^2^ = .0618, I^2^ = 65.88%; CG vs. CC + GG: Q = 54.40, df = 26, p-value = .0009, τ^2^ = .0293, I^2^ = 52.21%]. We also explored the sources of heterogeneity by the subgroup analysis based on cancer type and ethnic group. The results of our analysis suggested that the colon, lung, breast and liver cancers with the ethnic group of Asian and Caucasian were the main sources of heterogeneity of different studies (S1 Table).

#### IL-6 rs1800797 SNP

The finding of our analysis suggested that the IL-6 rs1800797 (-597G/A) polymorphism were not significantly associated with overall cancer risk under genetic models [A vs. G: OR = 0.99, 95% CI = 0.94–1.04, p-value = 0.7169; AA vs.GG: OR = 0.96, 95% CI = 0.85–1.08, p-value = 0.5152; AA + AG vs. GG: OR = 1.00, 95% CI = 0.93–1.08, p-value = 0.9289; AA vs. AG + GG: OR = 0.97, 95% CI = 0.87–1.07, p-value = 0.5064; AG vs. AA + GG: OR = 0.98, 95% CI = 0.91–1.05, p-value = 0.5025] (Table 4 and S1K–S1O Fig in S1 File).

The subgroup analysis based on cancer type showed that the blood, breast, colon, prostate and stomach cancers were not significantly associated with the IL-6 -597G/A polymorphism (Table 4). It also showed the significant role of IL-6 -597G/A polymorphism with the decreasing of cervical cancer risk under some genetic models [AA vs. GG: OR = 0.79, 95% CI = 0.63–0.98, p-value = 0.0390; AA vs. AG + GG: OR = 0.82, 95% CI = 0.68–1.00, p-value = 0.0474] and increasing of lung and skin cancer risks under the allelic model [A vs. G: OR = 1.19, 95% CI = 1.00–1.41, p-value = 0.0450] (Table 4).

The subgroup analysis based on ethnicity, the Asian, Caucasian and mixed population suggested that the IL-6 rs1800797 (-597G/A) polymorphism was not significantly associated with the overall cancer risk. Only for African population showed the significant association between this polymorphism and overall cancer risk by three genetic models [A vs. G: OR = 1.28, 95% CI = 0.98–1.67, p-value = 0.0191; AA + AG vs. GG: OR = 1.48, 95% CI = 1.08–2.03, p-value = 0.0135; AG vs. AA + GG: OR = 0.61, 95% CI = 0.44–0.84, p-value = 0.0010] (Table 4).

#### Source of heterogeneity

We found the insignificant heterogeneity of different studies in the analysis of IL-6 -597G/A polymorphism for overall cancer risk under the all genetic models. The subgroup analysis corresponding to cancer type and ethnic group were performed to observe the sources of heterogeneity. We found that only blood cancer was the main source of heterogeneity [A vs. G: Q = 6.56, df = 1, p-value = 0.0104, τ^2^ = 0.7679, I^2^ = 84.80%; AA + AG vs. GG: Q = 6.66, df = 1, p-value = 0.0099, τ^2^ = 0.8110, I^2^ = 85.00%] (S1 Table).

### Publication bias

**Fig 2:**
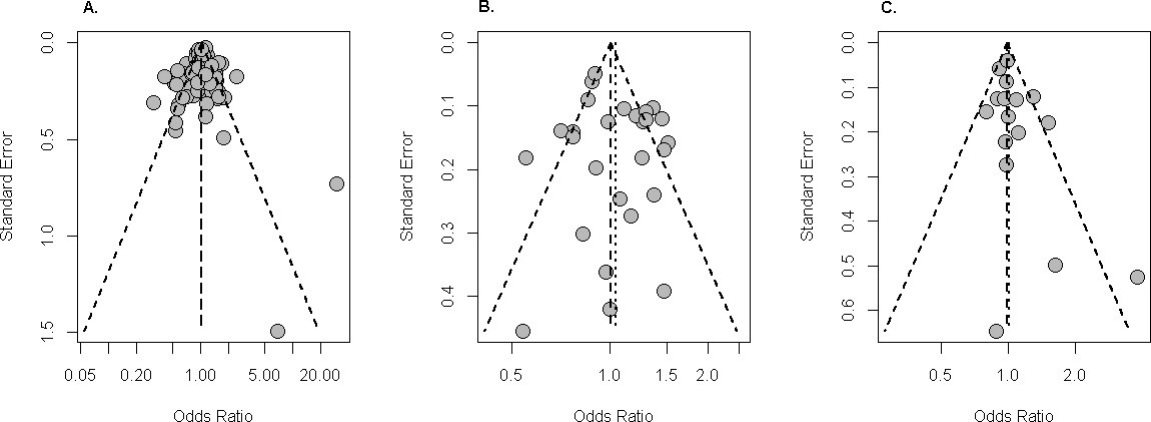
Funnel plot of the IL-6 polymorphisms to showing visual evidence of no publication bias. (A) -174G/C for C vs. G, (B) -572G/C for C vs. G and (C) -597G/A for A vs. G.

In this study the funnel plot was used to check the publication bias of IL-6 -174G/C and IL-6 -572G/C polymorphisms with the allelic model C versus G and IL-6 -597G/A polymorphism with the allelic model A versus G. According to the funnel plot, the distribution of ORs in terms of standard errors (SEs) was symmetric for each of three polymorphisms (-174G/C, -572G/C, -597G/A) and no publication bias was observed among the selected studies for this meta-analysis (Fig 2). Also, publication bias was checked through performing Begg’s test and Egger’s linear regression test. Results generated through both the Egger’s and Begg’s tests also suggested that there is no significant publication bias for the polymorphisms with the genetic models [C vs. G: p-value = 0.4778 (0.8030), and CC vs. GG: p-value = 0.5667 (0.7403) for the rs1800795 SNP; C vs. G: p-value = 0.3267 (0.6022) and CC vs. GG: p-value = 0.1664 (0.2347) for the rs1800796 SNP; A vs. G: p-value = 0.1175 (0.1768) and AA vs. GG: p-value = 0.6016 (0.7290) for the rs1800797 SNP] (see also S2A and S2B Table in S2 File). The p-value inside the first parenthesis was obtained by the Begg’s test.

### Sensitivity analysis

The sensitivity analysis was conducted to increase the reliability of this meta-analysis. First, the meta-analysis was conducted considering all studies. Then, the studies that did not pass the HWE test were removed and the meta-analysis was performed again using the reduced dataset of the respective genetic models. The analyzed results showed an insignificant change of association which suggested that the meta-analysis analysis data generated through this study is both stable and robust (see S2C–S2E Table in S2 File).

## Discussion and conclusion

In this paper we discussed the way of statistical modeling for meta-data analyses in details incorporating the goodness of fit test for checking the model adequacy. Then multi-case meta-analysis was conducted to find out the association of cancer risk with each of three SNPs (rs1800795, rs1800796, rs1800797) of the IL-6 gene. A total of 118 individual studies which included 50053 case and 65204 control samples, based on different cancers and ethnic groups were included in this extensive meta- analysis. The results computed through this study suggested that the IL-6 rs1800795 polymorphism is insignificantly associated with the overall cancer risk, but significantly reduced the risk of liver cancer under four genetic models (CC vs. GG; CC vs. CG + GG; CC + CG vs. GG; CG vs. CC + GG; C vs. G), which is in line with the previously reported multi-case meta-analysis in [130]. Also, this SNP showed significant association with the increasing risk of cervical and prostate cancers, where the results of cervical cancer are supported by the previous single-case meta-analysis in [123], but not with the multi-case meta-analysis in [130]. The results calculated for IL-6 rs1800796 polymorphism also showed significant association with overall cancer risk for one genetic model. This polymorphism showed significant association with the prostate and stomach cancers under four genetic models (CC vs. GG; CC vs. CG + GG; CC + CG vs. GG; C vs. G), where these results are supported by the previous multi-case meta-analysis in [130] and single-case meta-analysis in [111], respectively. Moreover, the results generated through this meta-analysis indicated that the rs1800796 polymorphism is significantly associated with the increasing risk of lung cancer. The IL-6 rs1800797 polymorphism analyzed data showed insignificant association with cancer risk, which is supported by previous single-case meta-analysis in [125]. Also, the results of this study showed the significant association of IL-6 rs1800797 polymorphism with increasing risk of cervical cancer, which showed insignificant association in [130].

The ethnicity based subgroup analysis data showed significant association between the rs1800795 polymorphism and the overall cancer risk of both African under three genetic models and Asian populations under four genetic models(CC vs. GG; CC vs. CG + GG; CC + CG vs. GG; C vs. G). For rs1800796 polymorphisms results suggested the significant association with the cancer risk of Asian populations. Also, the rs1800797 polymorphism was significantly associated with African ethnic groups for the cancer risk. All the results of subgroup analysis by ethnicity were supported by the previous multi-case meta-analysis in [130]. Thus, we observed that our multi-case meta-analysis results received more support than the previous multi-case meta-analysis results in [130] from the other single-case meta-analysis results in [123–129].

It should be mentioned here again that all of the previous meta-analyses [123–130] did not check the model adequacy through the goodness of fit test. To estimate the combined effects, all of them used fixed effect (FE) or random effect (RE) models based on Cochran’s homogeneity test though the sample sizes were small for some individual studies. For being small sample sizes, the individual effects may not be followed the normal distribution and the Cochran’s test may be produced misleading results about the homogeneity of individual effects. However, in our case, we used the GoF test suggested by Chen et al. [133] to fix the lack of model fitting. We observed that some of our fitted models contradict with the fitted models based on Cochran’s homogeneity test and significant changes in association between gene polymorphisms and cancer risks. In particularly, we observed the changes with some overall and subgroup cases of all polymorphisms (rs1800795, rs1800796, rs1800797). Due to the contradictory model selections, contradictory associations were also observed for three cases of rs1800795 polymorphism (liver cancer: CG vs. CC + GG; Asian ethnicity: CC + CG vs.GG and C vs. G) and single case of the rs1800796 polymorphism (stomach cancer: CC + CG vs. GG). However, there were some limitations on conducting this meta-analysis like for heterogeneity factors such as age, sex, family history, levels of IL-6 expression were not considered and that might affect the association. The literature reviewed and selected for this study was in English language only; therefore, the publication bias could not be completely avoided or some selection bias might occur. Also, the small sample size may affect the results for some types of cancer.

In conclusion, the results of this study indicated that the IL-6 gene is significantly associated with the overall cancer risk. Particularly, this gene showed significant association with 5 types of cancer risks (liver, prostate, cervical, stomach and lung) and insignificant association with 11 types of cancer risks (blood, breast, colon, neuroblastoma, oral, skin, thyroid, ovarian, pancreatic and renal cell carcinoma) by the sub-group analysis of cancer types. Comparative discussion showed that our current multi-case meta-analysis results received more support than any other individual previous meta-analysis results about the association between the IL-6 gene SNPs (rs1800795, rs1800796 and rs1800797) and different types of cancer risks. Therefore, the results generated through this detailed systematic meta-analysis based on larger sample size of the IL-6 gene polymorphisms provides more evidence for further exploring the IL-6 gene as a very potent prognostic biomarker for early detection of various types of cancers.

## Supporting information

S1 Table Heterogeneity analysis of IL-6 gene polymorphisms. (DOCX)

S1 File Forest plot of IL-6 gene polymorphisms (rs1800795, rs1800796, rs1800797) for five genetic models. (DOCX)

S2 File Egger’s linear regression and Begg’s test of IL-6 gene polymorphisms for checking the publication bias. (DOCX)

S3 File PRISMA checklist. (DOC)

S1 Data Full dataset of IL-6 gene rs1800795 polymorphism. (XLSX)

S2 Data Full dataset of IL-6 gene rs1800796 polymorphism. (XLSX)

S3 Data Full dataset of IL-6 gene rs1800797 polymorphism. (XLSX)
