# Supplementary material for: Statistical meta-analysis to investigate the association between the Interleukin-6 (IL-6) gene polymorphisms and cancer risk
Source: PLoS One. 2021 Mar 8;16(3):e0247055. doi: 10.1371/journal.pone.0247055 (PMC7939379; doi:10.1371/journal.pone.0247055)
Supplement: S1 Table — (DOCX) [file pone.0247055.s001.docx]

**S1 Table. Heterogeneity analysis of IL-6 gene polymorphisms**

| **rs1800795** | | | | | | | | | | | | | | | | | | | | | | | | | | | |
| --- | --- | --- | --- | --- | --- | --- | --- | --- | --- | --- | --- | --- | --- | --- | --- | --- | --- | --- | --- | --- | --- | --- | --- | --- | --- | --- | --- |
| **Overall Cancer** |  | | **Summary Measures** | | | | | | | | **Heterogeneity** | | | | | | | | | | **GoF test (p-val)** | | | | | | |
|  | Model | | OR | | 95% C.I. | | Z-val | | p-val | | Q-test | | d.f | | p-val | | Tau^2^ | | I^2 | | AD | | CvM | | SW | | |
| CC vs. GG | REM | | 1.06 | | [0.98; 1.16] | | 1.46 | | 0.1429 | | 258.44 | | 97 | | 0.0001 | | 0.0747 | | 62.47% | | 0.1237 | | 0.109 | | 0.16 | | |
| CC vs. CG + GG | REM | | 1.05 | | [0.98; 1.12] | | 1.27 | | 0.2054 | | 228.48 | | 97 | | 0.0001 | | 0.0478 | | 57.53% | | 0.118 | | 0.099 | | 0.065 | | |
| CC + CG vs. GG | FEM | | 1.02 | | [0.99; 1.05] | | 1.87 | | 0.062 | | 333.19 | | 100 | | 0.0001 | | 0.0485 | | 69.98% | | 0.0298 | | 0.026 | | 0.062 | | |
| CG vs. CC + GG | FEM | | 0.99 | | [0.96; 1.01] | | -1.11 | | 0.2681 | | 297.07 | | 100 | | 0.0001 | | 0.0385 | | 66.34% | | 0.0047 | | 0.002 | | 0.045 | | |
| C vs. G | REM | | 1.02 | | [0.97; 1.06] | | 0.76 | | 0.4459 | | 378.15 | | 100 | | 0.0001 | | 0.0290 | | 73.56% | | 0.3783 | | 0.35 | | 0.437 | | |
| **Cancer Type = Blood Cancer** |  | | **Summary Measures** | | | | | | | | **Heterogeneity** | | | | | | | | | | **GoF test (p-val)** | | | | | | |
|  | Model | | OR | | 95% C.I. | | Z-val | | p-val | | Q-test | | d.f | | p-val | | Tau^2^ | | I^2 | | AD | | CvM | | SW | | |
| CC vs. GG | REM | | 1.04 | | [0.86; 1.26] | | 0.49 | | 0.6246 | | 12.47 | | 11 | | 0.3295 | | 0.0132 | | 11.78% | | 0.9298 | | 0.884 | | 0.969 | | |
| CC vs. CG + GG | REM | | 1.06 | | [0.86; 1.29] | | 0.34 | | 0.6289 | | 14.06 | | 11 | | 0.0222 | | 0.0223 | | 21.73% | | 0.9391 | | 0.904 | | 0.972 | | |
| CC + CG vs. GG | REM | | 0.98 | | [0.87; 1.10] | | 0.04 | | 0.7399 | | 13.50 | | 12 | | 0.3338 | | 0.0051 | | 11.10% | | 0.3409 | | 0.377 | | 0.287 | | |
| CG vs. CC + GG | REM | | 0.99 | | [0.90; 1.08] | | -0.19 | | 0.7508 | | 12.26 | | 12 | | 0.4249 | | 0.0008 | | 2.13% | | 0.2262 | | 0.229 | | 0.215 | | |
| C vs. G | REM | | 1.01 | | [0.96; 1.07] | | -0.18 | | 0.8411 | | 19.12 | | 12 | | 0.0856 | | 0.0127 | | 37.25% | | 0.3949 | | 0.411 | | 0.362 | | |
| **Cancer Type = Breast Cancer** |  | | **Summary Measures** | | | | | | | | **Heterogeneity** | | | | | | | | | | **GoF test (p-val)** | | | | | | |
|  | Model | | OR | | 95% C.I. | | Z-val | | p-val | | Q-test | | d.f | | p-val | | Tau^2^ | | I^2 | | AD | | CvM | | SW | | |
| CC vs. GG | FEM | | 0.93 | | [0.84; 1.02] | | -1.50 | | 0.1329 | | 32.26 | | 13 | | 0.0026 | | 0.0683 | | 59.11% | | 0.0091 | | 0.01 | | 0.009 | | |
| CC vs. CG + GG | FEM | | 0.96 | | [0.88; 1.05] | | -0.83 | | 0.4042 | | 29.56 | | 13 | | 0.0088 | | 0.0494 | | 53.70% | | 0.0034 | | 0.003 | | 0.004 | | |
| CC + CG vs. GG | REM | | 0.95 | | [0.82; 1.11] | | -0.63 | | 0.5291 | | 41.96 | | 13 | | 0.0001 | | 0.0465 | | 69.02% | | 0.7037 | | 0.582 | | 0.788 | | |
| CG vs. CC + GG | FEM | | 0.98 | | [0.91; 1.04] | | -0.71 | | 0.4804 | | 88.94 | | 13 | | 0.0001 | | 0.1222 | | 85.38% | | 0.0164 | | 0.013 | | 0.049 | | |
| C vs. G | REM | | 1.00 | | [0.90; 1.11] | | 0.05 | | 0.9619 | | 39.53 | | 13 | | 0.0002 | | 0.0217 | | 67.11% | | 0.4011 | | 0.4 | | 0.41 | | |
| **Cancer Type = Cervical Cancer** |  | | **Summary Measures** | | | | | | | | **Heterogeneity** | | | | | | | | | | **GoF test (p-val)** | | | | | | |
|  | Model | | OR | | 95% C.I. | | Z-val | | p-val | | Q-test | | d.f | | p-val | | Tau^2^ | | I^2 | | AD | | CvM | | SW | | |
| CC vs. GG | REM | | 1.63 | | [1.06; 2.52] | | 2.22 | | 0.0266 | | 13.09 | | 6 | | 0.0420 | | 0.1571 | | 54.07% | | - | | - | | 0.5552 | | |
| CC vs. CG + GG | FEM | | 1.57 | | [1.25; 1.97] | | 3.93 | | 0.0001 | | 11.77 | | 6 | | 0.0676 | | 0.1151 | | 48.97% | | - | | - | | 0.6283 | | |
| CC + CG vs. GG | REM | | 1.31 | | [1.05; 1.64] | | 2.37 | | 0.0178 | | 12.77 | | 6 | | 0.0468 | | 0.0453 | | 53.02% | | - | | - | | 0.4887 | | |
| CG vs. CC + GG | REM | | 1.14 | | [0.89; 1.47] | | 1.05 | | 0.2927 | | 15.42 | | 6 | | 0.0172 | | 0.0647 | | 61.10% | | - | | - | | 0.7252 | | |
| C vs. G | REM | | 1.29 | | [1.07; 1.56] | | 2.67 | | 0.0075 | | 15.01 | | 6 | | 0.0202 | | 0.0354 | | 60.00% | | - | | - | | 0.229 | | |
| **Cancer Type = Colon Cancer** |  | | **Summary Measures** | | | | | | | | **Heterogeneity** | | | | | | | | | | **GoF test (p-val)** | | | | | | |
|  | Model | | OR | | 95% C.I. | | Z-val | | p-val | | Q-test | | d.f | | p-val | | Tau^2^ | | I^2 | | AD | | CvM | | SW | | |
| CC vs. GG | REM | | 0.98 | | [0.85; 1.13] | | -0.24 | | 0.8097 | | 28.22 | | 13 | | 0.0084 | | 0.0346 | | 53.94% | | 0.3247 | | 0.4 | | 0.24 | | |
| CC vs. CG + GG | REM | | 0.98 | | [0.87; 1.08] | | -0.82 | | 0.4136 | | 16.46 | | 13 | | 0.2251 | | 0.0065 | | 21.03% | | 0.3774 | | 0.326 | | 0.499 | | |
| CC + CG vs. GG | REM | | 1.02 | | [0.92; 1.14] | | 0.40 | | 0.6893 | | 39.22 | | 14 | | 0.0003 | | 0.0250 | | 64.30% | | 0.0548 | | 0.088 | | 0.031 | | |
| CG vs. CC + GG | FEM | | 1.02 | | [0.97; 1.08] | | 0.81 | | 0.4196 | | 17.01 | | 14 | | 0.2558 | | 0.0028 | | 17.68% | | 0.4232 | | 0.469 | | 0.495 | | |
| C vs. G | REM | | 1.01 | | [0.93; 1.09] | | 0.30 | | 0.7632 | | 43.03 | | 14 | | 0.0001 | | 0.0248 | | 67.46% | | 0.1184 | | 0.244 | | 0.041 | | |
| **Cancer Type = Liver Cancer** |  | | **Summary Measures** | | | | | | | | **Heterogeneity** | | | | | | | | | | **GoF test (p-val)** | | | | | | |
|  | Model | | OR | | 95% C.I. | | Z-val | | p-val | | Q-test | | d.f | | p-val | | Tau^2^ | | I^2 | | AD | | CvM | | SW | | |
| CC vs. GG | REM | | 0.61 | | [0.42; 0.88] | | -2.64 | | 0.0082 | | 0.77 | | 4 | | 0.9425 | | 0.0000 | | 0.00% | | - | | - | | 0.8028 | | |
| CC vs. CG + GG | REM | | 0.68 | | [0.48; 0.97] | | -2.14 | | 0.0321 | | 0.91 | | 4 | | 0.9232 | | 0.0000 | | 0.00% | | - | | - | | 0.5409 | | |
| CC + CG vs. GG | REM | | 0.69 | | [0.56; 0.85] | | -2.83 | | 0.0004 | | 6.14 | | 4 | | 0.1892 | | 0.0307 | | 34.81% | | - | | - | | 0.4969 | | |
| CG vs. CC + GG | REM | | 0.75 | | [0.54; 1.05] | | -1.69 | | 0.0998 | | 8.62 | | 4 | | 0.0714 | | 0.0744 | | 53.57% | | - | | - | | 0.4113 | | |
| C vs. G | REM | | 0.73 | | [0.62; 0.86] | | -3.02 | | 0.0001 | | 4.38 | | 4 | | 0.3570 | | 0.0036 | | 8.67% | | - | | - | | 0.922 | | |
| **Cancer Type = Lung Cancer** |  | | **Summary Measures** | | | | | | | | **Heterogeneity** | | | | | | | | | | **GoF test (p-val)** | | | | | | |
|  | Model | | OR | | 95% C.I. | | Z-val | | p-val | | Q-test | | d.f | | p-val | | Tau^2^ | | I^2 | | AD | | CvM | | SW | | |
| CC vs. GG | REM | | 1.08 | | [0.85; 1.36] | | 0.63 | | 0.5296 | | 8.37 | | 5 | | 0.1369 | | 0.0300 | | 40.27% | | - | | - | | 0.3199 | | |
| CC vs. CG + GG | REM | | 0.97 | | [0.76; 1.23] | | -0.26 | | 0.7981 | | 17.42 | | 5 | | 0.0038 | | 0.0516 | | 71.29% | | - | | - | | 0.3968 | | |
| CC + CG vs. GG | REM | | 1.08 | | [0.86; 1.84] | | 0.65 | | 0.5137 | | 10.36 | | 5 | | 0.0656 | | 0.0332 | | 51.74% | | - | | - | | 0.076 | | |
| CG vs. CC + GG | REM | | 1.07 | | [0.86; 1.34] | | 0.62 | | 0.5334 | | 18.98 | | 5 | | 0.0019 | | 0.0464 | | 73.66% | | - | | - | | 0.1036 | | |
| C vs. G | REM | | 1.01 | | [0.87; 1.18] | | 0.11 | | 0.9102 | | 16.80 | | 5 | | 0.0049 | | 0.0215 | | 70.23% | | - | | - | | 0.964 | | |
| **Cancer Type = Neuroblastoma (NB)** |  | | **Summary Measures** | | | | | | | | **Heterogeneity** | | | | | | | | | | **GoF test (p-val)** | | | | | | |
|  | Model | | OR | | 95% C.I. | | Z-val | | p-val | | Q-test | | d.f | | p-val | | Tau^2^ | | I^2 | | AD | | CvM | | SW | | |
| CC vs. GG | FEM | | 1.18 | | [0.71; 1.95] | | 0.64 | | 0.5244 | | 0.76 | | 1 | | 0.3832 | | 0.0000 | | 0.00% | | - | | - | | - | | |
| CC vs. CG + GG | FEM | | 1.15 | | [0.71; 1.88] | | 0.56 | | 0.5731 | | 0.41 | | 1 | | 0.5244 | | 0.0000 | | 0.00% | | - | | - | | - | | |
| CC + CG vs. GG | FEM | | 1.09 | | [0.84; 1.41] | | 0.63 | | 0.5256 | | 1.50 | | 1 | | 0.2209 | | 0.0330 | | 33.26% | | - | | - | | - | | |
| CG vs. CC + GG | FEM | | 1.05 | | [0.80; 1.37] | | 0.35 | | 0.7259 | | 0.87 | | 1 | | 0.3520 | | 0.0000 | | 0.00% | | - | | - | | - | | |
| C vs. G | FEM | | 1.08 | | [0.88; 1.33] | | 0.75 | | 0.4570 | | 1.57 | | 1 | | 0.2108 | | 0.0233 | | 36.13% | | - | | - | | - | | |
| **Cancer Type = Oral Cancer** |  | | **Summary Measures** | | | | | | | | **Heterogeneity** | | | | | | | | | | **GoF test (p-val)** | | | | | | |
|  | Model | | OR | | 95% C.I. | | Z-val | | p-val | | Q-test | | d.f | | p-val | | Tau^2^ | | I^2 | | AD | | CvM | | SW | | |
| CC vs. GG | REM | | 0.69 | | [0.08; 6.27] | | -0.33 | | 0.7430 | | 30.33 | | 2 | | 0.0001 | | 3.5592 | | 93.39% | | - | | - | | - | | |
| CC vs. CG + GG | REM | | 0.74 | | [0.18; 3.04] | | -0.42 | | 0.6714 | | 13.46 | | 2 | | 0.0012 | | 1.3424 | | 85.11% | | - | | - | | - | | |
| CC + CG vs. GG | REM | | 0.80 | | [0.16; 4.09] | | -0.27 | | 0.7864 | | 68.16 | | 2 | | 0.0001 | | 2.0281 | | 97.06% | | - | | - | | - | | |
| CG vs. CC + GG | REM | | 0.86 | | [0.25; 2.95] | | -0.23 | | 0.8160 | | 39.32 | | 2 | | 0.0001 | | 1.1177 | | 94.91% | | - | | - | | - | | |
| C vs. G | REM | | 0.81 | | [0.25; 2.58] | | -0.36 | | 0.7197 | | 60.31 | | 2 | | 0.0001 | | 1.0154 | | 96.68% | | - | | - | | - | | |
| **Cancer Type = Prostate Cancer** |  | | **Summary Measures** | | | | | | | | **Heterogeneity** | | | | | | | | | | **GoF test (p-val)** | | | | | | |
|  | Model | | OR | | 95% C.I. | | Z-val | | p-val | | Q-test | | d.f | | p-val | | Tau^2^ | | I^2 | | AD | | CvM | | SW | | |
| CC vs. GG | REM | | 1.24 | | [1.05; 1.46] | | 2.59 | | 0.0096 | | 31.59 | | 13 | | 0.0028 | | 0.0431 | | 58.85% | | 0.0607 | | 0.061 | | 0.061 | | |
| CC vs. CG + GG | REM | | 1.19 | | [1.03; 1.39] | | 2.32 | | 0.0202 | | 33.78 | | 13 | | 0.0013 | | 0.0373 | | 61.52% | | 0.1108 | | 0.132 | | 0.09 | | |
| CC + CG vs. GG | REM | | 1.09 | | [0.98; 1.22] | | 1.58 | | 0.1142 | | 29.02 | | 13 | | 0.0065 | | 0.0188 | | 55.21% | | 0.8771 | | 0.926 | | 0.722 | | |
| CG vs. CC + GG | REM | | 0.97 | | [0.91; 1.05] | | -0.71 | | 0.4773 | | 15.80 | | 13 | | 0.2603 | | 0.0030 | | 17.70% | | 0.6794 | | 0.579 | | 0.664 | | |
| C vs. G | REM | | 1.09 | | [1.00; 1.19] | | 2.01 | | 0.0441 | | 40.21 | | 13 | | 0.0001 | | 0.0149 | | 67.67% | | 0.1623 | | 0.131 | | 0.229 | | |
| **Cancer Type = Skin Cancer** |  | | **Summary Measures** | | | | | | | | **Heterogeneity** | | | | | | | | | | **GoF test (p-val)** | | | | | | |
|  | Model | | OR | | 95% C.I. | | Z-val | | p-val | | Q-test | | d.f | | p-val | | Tau^2^ | | I^2 | | AD | | CvM | | SW | | |
| CC vs. GG | REM | | 0.99 | | [0.75; 1.32] | | -0.04 | | 0.9680 | | 0.96 | | 3 | | 0.8109 | | 0.0000 | | 0.00% | | - | | - | | 0.0828 | | |
| CC vs. CG + GG | REM | | 0.96 | | [0.76; 1.20] | | -0.45 | | 0.6564 | | 0.51 | | 3 | | 0.9170 | | 0.0000 | | 0.00% | | - | | - | | 0.2467 | | |
| CC + CG vs. GG | REM | | 1.05 | | [0.84; 1.31] | | 0.41 | | 0.6808 | | 1.54 | | 3 | | 0.6723 | | 0.0000 | | 0.00% | | - | | - | | 0.6633 | | |
| CG vs. CC + GG | REM | | 1.08 | | [0.88; 1.31] | | 0.74 | | 0.4597 | | 0.96 | | 3 | | 0.8105 | | 0.0000 | | 0.00% | | - | | - | | 0.1051 | | |
| C vs. G | REM | | 1.00 | | [0.87; 1.15] | | -0.01 | | 0.9912 | | 1.25 | | 3 | | 0.7406 | | 0.0000 | | 0.00% | | - | | - | | 0.108 | | |
| **Cancer Type = Stomach Cancer** |  | | **Summary Measures** | | | | | | | | **Heterogeneity** | | | | | | | | | | **GoF test (p-val)** | | | | | | |
|  | Model | | OR | | 95% C.I. | | Z-val | | p-val | | Q-test | | d.f | | p-val | | Tau^2^ | | I^2 | | AD | | CvM | | SW | | |
| CC vs. GG | REM | | 1.12 | | [0.85; 1.50] | | 0.83 | | 0.4087 | | 15.60 | | 11 | | 0.1588 | | 0.0683 | | 29.25% | | 0.3252 | | 0.274 | | 0.366 | | |
| CC vs. CG + GG | REM | | 1.07 | | [0.81; 1.40] | | 0.47 | | 0.6405 | | 16.70 | | 11 | | 0.1195 | | 0.0688 | | 33.84% | | 0.4832 | | 0.41 | | 0.558 | | |
| CC + CG vs. GG | FEM | | 1.08 | | [0.92; 1.22] | | 0.79 | | 0.4317 | | 63.06 | | 12 | | 0.0001 | | 0.3244 | | 80.74% | | 0.0141 | | 0.022 | | 0.007 | | |
| CG vs. CC + GG | FEM | | 1.04 | | [0.90; 1.19] | | 0.52 | | 0.6024 | | 61.98 | | 12 | | 0.0001 | | 0.3021 | | 80.44% | | 0.0084 | | 0.014 | | 0.003 | | |
| C vs. G | FEM | | 1.04 | | [0.94; 1.16] | | 0.43 | | 0.4517 | | 55.80 | | 12 | | 0.0001 | | 0.1424 | | 78.38% | | 0.0077 | | 0.009 | | 0.005 | | |
| **Cancer Type = Thyroid Cancer** |  | | **Summary Measures** | | | | | | | | **Heterogeneity** | | | | | | | | | | **GoF test (p-val)** | | | | | | |
|  | Model | | OR | | 95% C.I. | | Z-val | | p-val | | Q-test | | d.f | | p-val | | Tau^2^ | | I^2 | | AD | | CvM | | SW | | |
| CC vs. GG | FEM | | 1.20 | | [0.67; 2.13] | | 0.62 | | 0.5364 | | 1.10 | | 1 | | 0.2943 | | 0.0190 | | 9.07% | | - | | - | | - | | |
| CC vs. CG + GG | FEM | | 1.30 | | [0.75; 2.27] | | 0.94 | | 0.3477 | | 0.93 | | 1 | | 0.3374 | | 0.0000 | | 0.00% | | - | | - | | - | | |
| CC + CG vs. GG | FEM | | 0.87 | | [0.63; 1.21] | | -0.82 | | 0.4149 | | 0.66 | | 1 | | 0.4171 | | 0.0000 | | 0.00% | | - | | - | | - | | |
| CG vs. CC + GG | FEM | | 0.79 | | [0.57; 1.10] | | -1.38 | | 0.1680 | | 0.08 | | 1 | | 0.7779 | | 0.0000 | | 0.00% | | - | | - | | - | | |
| C vs. G | FEM | | 0.97 | | [0.76; 1.25] | | -0.21 | | 0.8399 | | 1.16 | | 1 | | 0.2810 | | 0.0060 | | 13.95% | | - | | - | | - | | |
| **Cancer Type = Other Cancers** |  | | **Summary Measures** | | | | | | | | **Heterogeneity** | | | | | | | | | | **GoF test (p-val)** | | | | | | |
|  | Model | | OR | | 95% C.I. | | Z-val | | p-val | | Q-test | | d.f | | p-val | | Tau^2^ | | I^2 | | AD | | CvM | | SW | | |
| CC vs. GG | FEM | | 1.10 | | [0.58; 2.10] | | 0.29 | | 0.7683 | | 3.67 | | 2 | | 0.1832 | | 0.3947 | | 41.07% | | - | | - | | 0.0092 | | |
| CC vs. CG + GG | FEM | | 1.09 | | [0.73; 1.61] | | 0.41 | | 0.6802 | | 3.44 | | 2 | | 0.1929 | | 0.1665 | | 39.24% | | - | | - | | 0.0124 | | |
| CC + CG vs. GG | FEM | | 1.29 | | [0.80; 2.13] | | 0.99 | | 0.3236 | | 1.14 | | 2 | | 0.5664 | | 0.0000 | | 0.00% | | - | | - | | 0.0010 | | |
| CG vs. CC + GG | FEM | | 1.07 | | [0.74; 1.56] | | 0.37 | | 0.7148 | | 2.15 | | 2 | | 0.3412 | | 0.0097 | | 7.00% | | - | | - | | 0.911 | | |
| C vs. G | FEM | | 1.14 | | [0.85; 1.54] | | -0.86 | | 0.3711 | | 3.80 | | 2 | | 0.1498 | | 0.0688 | | 47.33% | | - | | - | | 0.113 | | |
| **Ethnicity = African** |  | | **Summary Measures** | | | | | | | | **Heterogeneity** | | | | | | | | | | **GoF test (p-val)** | | | | | | |
|  | Model | | OR | | 95% C.I. | | Z-val | | p-val | | Q-test | | d.f | | p-val | | Tau^2^ | | I^2 | | AD | | CvM | | SW | | |
| CC vs. GG | FEM | | 1.58 | | [0.68; 3.66] | | 1.05 | | 0.2895 | | 0.01 | | 1 | | 0.9209 | | 0.0000 | | 0.00% | | - | | - | | - | | |
| CC vs. CG + GG | FEM | | 1.40 | | [0.61; 3.25] | | 0.79 | | 0.4248 | | 0.02 | | 1 | | 0.8870 | | 0.0000 | | 0.00% | | - | | - | | - | | |
| CC + CG vs. GG | FEM | | 1.66 | | [1.20; 2.3] | | 3.09 | | 0.0027 | | 0.96 | | 2 | | 0.6191 | | 0.0000 | | 0.00% | | - | | - | | - | | |
| CG vs. CC + GG | FEM | | 1.64 | | [1.17; 2.30] | | 2.90 | | 0.0037 | | 0.98 | | 2 | | 0.6153 | | 0.0000 | | 0.00% | | - | | - | | - | | |
| C vs. G | FEM | | 1.54 | | [1.16; 2.04] | | 2.97 | | 0.0030 | | 1.07 | | 2 | | 0.5876 | | 0.0000 | | 0.00% | | - | | - | | - | | |
| **Ethnicity = Asian** |  | | **Summary Measures** | | | | | | | | **Heterogeneity** | | | | | | | | | | **GoF test (p-val)** | | | | | | |
|  | Model | | OR | | 95% C.I. | | Z-val | | p-val | | Q-test | | d.f | | p-val | | Tau^2^ | | I^2 | | AD | | CvM | | SW | | |
| CC vs. GG | REM | | 1.56 | | [1.19; 2.03] | | 3.28 | | 0.0011 | | 28.15 | | 15 | | 0.0237 | | 0.1095 | | 45.79% | | 0.1667 | | 0.192 | | 0.109 | | |
| CC vs. CG + GG | FEM | | 1.37 | | [1.22; 1.53] | | 3.42 | | 0.0001 | | 46.90 | | 15 | | 0.0001 | | 0.1676 | | 66.98% | | 0.0369 | | 0.037 | | 0.023 | | |
| CC + CG vs. GG | FEM | | 1.17 | | [1.08; 1.29] | | 3.03 | | 0.0024 | | 73.89 | | 16 | | 0.0001 | | 0.1736 | | 78.26% | | 0.0019 | | 0.004 | | 4E-04 | | |
| CG vs. CC + GG | FEM | | 0.93 | | [0.85; 1.02] | | -1.58 | | 0.1151 | | 122.40 | | 16 | | 0.0001 | | 0.2668 | | 86.91% | | 0.0013 | | 0.001 | | 0.001 | | |
| C vs. G | FEM | | 1.20 | | [1.12; 1.29] | | 5.32 | | 0.0000 | | 74.80 | | 16 | | 0.0001 | | 0.0834 | | 78.58% | | 0.0053 | | 0.007 | | 0.002 | | |
| **Ethnicity = Caucasian** |  | | **Summary Measures** | | | | | | | | **Heterogeneity** | | | | | | | | | | **GoF test (p-val)** | | | | | | |
|  | Model | | OR | | 95% C.I. | | Z-val | | p-val | | Q-test | | d.f | | p-val | | Tau^2^ | | I^2 | | AD | | CvM | | SW | | |
| CC vs. GG | REM | | 1.01 | | [0.92; 1.11] | | 0.23 | | 0.8193 | | 143.32 | | 64 | | 0.0001 | | 0.0561 | | 55.34% | | 0.1615 | | 0.134 | | 0.328 | | |
| CC vs. CG + GG | REM | | 1.00 | | [0.94; 1.07] | | 0.07 | | 0.9469 | | 104.30 | | 64 | | 0.0011 | | 0.0226 | | 38.63% | | 0.292 | | 0.222 | | 0.425 | | |
| CC + CG vs. GG | FEM | | 1.00 | | [0.97; 1.04] | | 0.15 | | 0.8788 | | 194.68 | | 65 | | 0.0001 | | 0.0443 | | 66.61% | | 0.0405 | | 0.029 | | 0.109 | | |
| CG vs. CC + GG | FEM | | 1.00 | | [0.97; 1.04] | | 0.10 | | 0.9164 | | 122.32 | | 65 | | 0.0001 | | 0.0179 | | 46.86% | | 0.0049 | | 0.004 | | 0.026 | | |
| C vs. G | REM | | 1.00 | | [0.95; 1.05] | | 0.09 | | 0.9291 | | 204.61 | | 65 | | 0.0001 | | 0.0228 | | 68.23% | | 0.0552 | | 0.031 | | 0.24 | | |
| **Ethnicity = Mixed** |  | | **Summary Measures** | | | | | | | | **Heterogeneity** | | | | | | | | | | **GoF test (p-val)** | | | | | | |
|  | Model | | OR | | 95% C.I. | | Z-val | | p-val | | Q-test | | d.f | | p-val | | Tau^2^ | | I^2 | | AD | | CvM | | SW | | |
| CC vs. GG | REM | | 0.97 | | [0.74; 1.27] | | -0.22 | | 0.8224 | | 55.77 | | 14 | | 0.0001 | | 0.1122 | | 74.89% | | 0.3719 | | 0.381 | | 0.306 | | |
| CC vs. CG + GG | REM | | 1.02 | | [0.80; 1.31] | | 0.17 | | 0.8629 | | 55.77 | | 14 | | 0.0001 | | 0.0979 | | 74.89% | | 0.724 | | 0.703 | | 0.67 | | |
| CC + CG vs. GG | REM | | 0.89 | | [0.77; 1.04] | | -1.44 | | 0.1493 | | 50.39 | | 14 | | 0.0001 | | 0.0390 | | 72.21% | | 0.7972 | | 0.833 | | 0.707 | | |
| CG vs. CC + GG | REM | | 0.89 | | [0.78; 1.03] | | -1.58 | | 0.1134 | | 38.44 | | 14 | | 0.0004 | | 0.0283 | | 63.58% | | 0.2463 | | 0.221 | | 0.419 | | |
| C vs. G | REM | | 0.93 | | [0.82; 1.06] | | -1.11 | | 0.2676 | | 66.73 | | 14 | | 0.0001 | | 0.0330 | | 79.02% | | 0.5113 | | 0.67 | | 0.297 | | |
| **rs1800796** | | | | | | | | | | | | | | | | | | | | | | | | | | | |
| **Cancer Type = Overall Cancers** | |  | | **Summary Measures** | | | | | | | | **Heterogeneity** | | | | | | | | | | **GoF test (p-val)** | | | | | |
|  | | Model | | OR | | 95% C.I. | | Z-val | | p-val | | Q-test | | d.f | | p-val | | Tau^2^ | | I^2 | | AD | | CvM | | SW | |
| CC vs. GG | | REM | | 1.03 | | [0.85; 1.25] | | 0.30 | | 0.7635 | | 55.82 | | 26 | | 0.0006 | | 0.1033 | | 53.41% | | 0.9595 | | 0.909 | | 0.992 | |
| CC vs. CG + GG | | REM | | 0.99 | | [0.86; 1.14] | | -0.18 | | 0.8582 | | 49.23 | | 26 | | 0.0039 | | 0.0459 | | 47.18% | | 0.965 | | 0.959 | | 0.9690 | |
| CC + CG vs. GG | | REM | | 1.07 | | [0.94; 1.21] | | 1.05 | | 0.2931 | | 76.19 | | 26 | | 0.0001 | | 0.0618 | | 65.88% | | 0.996 | | 0.909 | | 0.992 | |
| CG vs. CC + GG | | REM | | 1.12 | | [1.01; 1.23] | | 2.19 | | 0.0288 | | 54.40 | | 26 | | 0.0009 | | 0.0293 | | 52.21% | | 0.1852 | | 0.203 | | 0.21 | |
| C vs. G | | REM | | 1.04 | | [0.95; 1.15] | | 0.87 | | 0.3839 | | 89.96 | | 26 | | 0.0001 | | 0.0391 | | 71.07% | | 0.3656 | | 0.472 | | 0.228 | |
| **Cancer Type = Colon Cancers** | |  | | **Summary Measures** | | | | | | | | **Heterogeneity** | | | | | | | | | | **GoF test (p-val)** | | | | | |
|  | | Model | | OR | | 95% C.I. | | Z-val | | p-val | | Q-test | | d.f | | p-val | | Tau^2^ | | I^2 | | AD | | CvM | | SW | |
| CC vs. GG | | REM | | 1.04 | | [0.67; 1.64] | | 0.19 | | 0.8507 | | 6.75 | | 2 | | 0.0342 | | 0.0916 | | 70.38% | | - | | - | | 0.5192 | |
| CC vs. CG + GG | | REM | | 1.05 | | [0.73; 1.50] | | 0.25 | | 0.8000 | | 4.89 | | 2 | | 0.0866 | | 0.0532 | | 59.12% | | - | | - | | 0.5192 | |
| CC + CG vs. GG | | REM | | 1.04 | | [0.67; 1.63] | | 0.20 | | 0.8507 | | 9.87 | | 2 | | 0.0072 | | 0.0705 | | 79.74% | | - | | - | | 0.7051 | |
| CG vs. CC + GG | | REM | | 1.07 | | [0.85; 1.36] | | 0.59 | | 0.5552 | | 3.47 | | 2 | | 0.1761 | | 0.0197 | | 42.43% | | - | | - | | 0.8369 | |
| C vs. G | | REM | | 1.10 | | [0.79; 1.53] | | 0.58 | | 0.5613 | | 13.42 | | 2 | | 0.0012 | | 0.0646 | | 85.09% | | - | | - | | 0.723 | |
| **Cancer Type = Lung Cancers** | |  | | **Summary Measures** | | | | | | | | **Heterogeneity** | | | | | | | | | | **GoF test (p-val)** | | | | | |
|  | | Model | | OR | | 95% C.I. | | Z-val | | p-val | | Q-test | | d.f | | p-val | | Tau^2^ | | I^2 | | AD | | CvM | | SW | |
| CC vs. GG | | REM | | 1.13 | | [0.75; 1.69] | | 0.59 | | 0.5575 | | 12.78 | | 6 | | 0.0469 | | 0.1477 | | 53.00% | | - | | - | | 0.3382 | |
| CC vs. CG + GG | | FEM | | 0.93 | | [0.74; 1.17] | | -0.62 | | 0.5400 | | 10.58 | | 6 | | 0.1026 | | 0.0777 | | 43.24% | | - | | - | | 0.1614 | |
| CC + CG vs. GG | | REM | | 1.31 | | [1.04; 1.65] | | 2.28 | | 0.0228 | | 18.77 | | 6 | | 0.0046 | | 0.0604 | | 68.04% | | - | | - | | 0.7120 | |
| CG vs. CC + GG | | REM | | 1.31 | | [1.08; 1.59] | | 2.69 | | 0.0072 | | 14.24 | | 6 | | 0.0271 | | 0.0383 | | 57.87% | | - | | - | | 0.4126 | |
| C vs. G | | REM | | 1.19 | | [0.98; 1.43] | | 1.79 | | 0.0734 | | 20.36 | | 6 | | 0.0024 | | 0.0423 | | 70.53% | | - | | - | | 0.224 | |
| **Cancer Type = Prostate Cancers** | |  | | **Summary Measures** | | | | | | | | **Heterogeneity** | | | | | | | | | | **GoF test (p-val)** | | | | | |
|  | | Model | | OR | | 95% C.I. | | Z-val | | p-val | | Q-test | | d.f | | p-val | | Tau^2^ | | I^2 | | AD | | CvM | | SW | |
| CC vs. GG | | REM | | 0.52 | | [0.37; 0.72] | | -3.90 | | 0.0001 | | 2.98 | | 5 | | 0.7025 | | 0.0000 | | 0.00% | | - | | - | | 0.4301 | |
| CC vs. CG + GG | | REM | | 0.67 | | [0.53; 0.84] | | -3.50 | | 0.0005 | | 4.44 | | 5 | | 0.4882 | | 0.0000 | | 0.00% | | - | | - | | 0.8184 | |
| CC + CG vs. GG | | REM | | 0.74 | | [0.61; 0.90] | | -3.02 | | 0.0025 | | 4.50 | | 5 | | 0.4798 | | 0.0000 | | 0.00% | | - | | - | | 0.4851 | |
| CG vs. CC + GG | | REM | | 1.00 | | [0.84; 1.18] | | -0.02 | | 0.9811 | | 4.64 | | 5 | | 0.4608 | | 0.0000 | | 0.00% | | - | | - | | 0.4505 | |
| C vs. G | | REM | | 0.74 | | [0.64; 0.85] | | -4.21 | | 0.0000 | | 5.23 | | 5 | | 0.3881 | | 0.0014 | | 4.46% | | - | | - | | 0.7668 | |
| **Cancer Type = Stomach Cancers** | |  | | **Summary Measures** | | | | | | | | **Heterogeneity** | | | | | | | | | | **GoF test (p-val)** | | | | | |
|  | | Model | | OR | | 95% C.I. | | Z-val | | p-val | | Q-test | | d.f | | p-val | | Tau^2^ | | I^2 | | AD | | CvM | | SW | |
| CC vs. GG | | REM | | 1.41 | | [1.10; 1.81] | | 2.62 | | 0.0076 | | 6.16 | | 8 | | 0.6291 | | 0.0000 | | 0.00% | | 0.4019 | | 0.437 | | 0.325 | |
| CC vs. CG + GG | | REM | | 1.29 | | [1.07; 1.55] | | 2.62 | | 0.0080 | | 6.35 | | 8 | | 0.6084 | | 0.0000 | | 0.00% | | 0.8544 | | 0.825 | | 0.918 | |
| CC + CG vs. GG | | REM | | 1.41 | | [1.09; 1.81] | | 2.61 | | 0.0088 | | 14.29 | | 8 | | 0.0746 | | 0.0510 | | 44.00% | | 0.8239 | | 0.785 | | 0.909 | |
| CG vs. CC + GG | | REM | | 1.02 | | [0.79; 1.31] | | 0.13 | | 0.8940 | | 18.79 | | 8 | | 0.0160 | | 0.0708 | | 57.42% | | 0.3595 | | 0.282 | | 0.5820 | |
| C vs. G | | REM | | 1.16 | | [1.03; 1.30] | | 2.52 | | 0.0069 | | 8.59 | | 8 | | 0.3777 | | 0.0023 | | 6.92% | | 0.2727 | | 0.361 | | 0.187 | |
| **Cancer Type = Other Cancers** | |  | | **Summary Measures** | | | | | | | | **Heterogeneity** | | | | | | | | | | **GoF test (p-val)** | | | | | |
|  | | Model | | OR | | 95% C.I. | | Z-val | | p-val | | Q-test | | d.f | | p-val | | Tau^2^ | | I^2 | | AD | | CvM | | SW | |
| CC vs. GG | | REM | | 1.13 | | [0.47; 2.68] | | 0.27 | | 0.7852 | | 4.51 | | 1 | | 0.0338 | | 0.3124 | | 77.80% | | - | | - | | - | |
| CC vs. CG + GG | | FEM | | 0.90 | | [0.78; 1.04] | | -1.49 | | 0.1400 | | 3.12 | | 1 | | 0.0773 | | 0.1651 | | 67.96% | | - | | - | | - | |
| CC + CG vs. GG | | REM | | 1.03 | | [0.64; 1.67] | | 0.14 | | 0.8922 | | 5.31 | | 1 | | 0.0212 | | 0.0964 | | 81.16% | | - | | - | | - | |
| CG vs. CC + GG | | FEM | | 1.12 | | [0.99; 1.28] | | 1.73 | | 0.0841 | | 0.44 | | 1 | | 0.5083 | | 0.0000 | | 0.00% | | - | | - | | - | |
| C vs. G | | REM | | 1.05 | | [0.72; 1.53] | | 0.27 | | 0.7878 | | 7.94 | | 1 | | 0.0048 | | 0.0640 | | 87.41% | | - | | - | | - | |
| **Ethnicity = Asian** | |  | | **Summary Measures** | | | | | | | | **Heterogeneity** | | | | | | | | | | **GoF test (p-val)** | | | | | |
|  | | Model | | OR | | 95% C.I. | | Z-val | | p-val | | Q-test | | d.f | | p-val | | Tau^2^ | | I^2 | | AD | | CvM | | SW | |
| CC vs. GG | | REM | | 1.02 | | [0.79; 1.31] | | 0.15 | | 0.8812 | | 43.49 | | 17 | | 0.0004 | | 0.1579 | | 60.90% | | 0.5528 | | 0.44 | | 0.644 | |
| CC vs. CG + GG | | REM | | 1.00 | | [0.83; 1.20] | | -0.05 | | 0.9636 | | 40.00 | | 17 | | 0.0013 | | 0.0687 | | 57.49% | | 0.7069 | | 0.694 | | 0.761 | |
| CC + CG vs. GG | | REM | | 1.06 | | [0.91; 1.25] | | 0.75 | | 0.4557 | | 50.99 | | 17 | | 0.0001 | | 0.0705 | | 66.66% | | 0.5789 | | 0.57 | | 0.585 | |
| CG vs. CC + GG | | REM | | 1.13 | | [1.01; 1.27] | | 2.18 | | 0.0293 | | 33.74 | | 17 | | 0.0091 | | 0.0260 | | 49.61% | | 0.1177 | | 0.143 | | 0.081 | |
| C vs. G | | REM | | 1.04 | | [0.92; 1.19] | | 0.68 | | 0.4974 | | 69.20 | | 17 | | 0.0001 | | 0.0496 | | 75.43% | | 0.3495 | | 0.442 | | 0.234 | |
| **Ethnicity = Caucasian** | |  | | **Summary Measures** | | | | | | | | **Heterogeneity** | | | | | | | | | | **GoF test (p-val)** | | | | | |
|  | | Model | | OR | | 95% C.I. | | Z-val | | p-val | | Q-test | | d.f | | p-val | | Tau^2^ | | I^2 | | AD | | CvM | | SW | |
| CC vs. GG | | REM | | 1.07 | | [0.76; 1.49] | | 0.37 | | 0.7105 | | 11.37 | | 7 | | 0.1234 | | 0.0629 | | 38.41% | | - | | - | | 0.6254 | |
| CC vs. CG + GG | | REM | | 0.97 | | [0.78; 1.21] | | -0.26 | | 0.7964 | | 8.14 | | 7 | | 0.3208 | | 0.0144 | | 13.95% | | - | | - | | 0.9707 | |
| CC + CG vs. GG | | REM | | 1.10 | | [0.87; 1.39] | | 0.77 | | 0.4398 | | 21.33 | | 7 | | 0.0033 | | 0.0636 | | 67.18% | | - | | - | | 0.4153 | |
| CG vs. CC + GG | | REM | | 1.12 | | [0.89; 1.40] | | 0.96 | | 0.3391 | | 17.08 | | 7 | | 0.0169 | | 0.0513 | | 59.03% | | - | | - | | 0.2701 | |
| C vs. G | | REM | | 1.04 | | [0.87; 1.26] | | 0.46 | | 0.6445 | | 19.46 | | 7 | | 0.0069 | | 0.0366 | | 64.03% | | - | | - | | 0.6807 | |
| **Ethnicity = Mixed** | |  | | **Summary Measures** | | | | | | | | **Heterogeneity** | | | | | | | | | | **GoF test (p-val)** | | | | | |
|  | | Model | | OR | | 95% C.I. | | Z-val | | p-val | | Q-test | | d.f | | p-val | | Tau^2^ | | I^2 | | AD | | CvM | | SW | |
| CC vs. GG | | FEM | | 3.20 | | [0.28; 36.45] | | 0.94 | | 0.3487 | | 0.00 | | 0 | | NA | | 0.0000 | | 0.00% | | - | | - | | - | |
| CC vs. CG + GG | | FEM | | 3.44 | | [0.30; 38.90] | | 1.00 | | 0.3181 | | 0.00 | | 0 | | NA | | 0.0000 | | 0.00% | | - | | - | | - | |
| CC + CG vs. GG | | FEM | | 0.83 | | [0.37; 1.86] | | -0.44 | | 0.6590 | | 0.00 | | 0 | | NA | | 0.0000 | | 0.00% | | - | | - | | - | |
| CG vs. CC + GG | | FEM | | 0.70 | | [0.30; 1.63] | | -0.82 | | 0.4130 | | 0.00 | | 0 | | NA | | 0.0000 | | 0.00% | | - | | - | | - | |
| C vs. G | | FEM | | 0.97 | | [0.48; 1.98] | | -0.36 | | 0.7169 | | 0.00 | | 0 | | NA | | 0.0000 | | 0.00% | | - | | - | | - | |
| **rs1800797** | | | | | | | | | | | | | | | | | | | | | | | | | | | |
| **Cancer Type = Overall Cancers** | |  | | **Summary Measures** | | | | | | | | **Heterogeneity** | | | | | | | | | | **GoF test (p-vall)** | | | | | |
|  | | Model | | OR | | 95% C.I. | | Z-val | | p-vall | | Q-test | | d.f | | p-val | | Tau^2^ | | I^2 | | AD | | CvM | | | SW |
| AA vs. GG | | REM | | 0.96 | | [0.85; 1.08] | | -0.65 | | 0.5152 | | 15.06 | | 14 | | 0.3742 | | 0.0040 | | 7.00% | | 0.7936 | | 0.834 | | | 0.696 |
| AA vs. AG + GG | | REM | | 0.97 | | [0.87; 1.07] | | -0.66 | | 0.5046 | | 14.45 | | 14 | | 0.4164 | | 0.0014 | | 3.00% | | 0.7424 | | 0.771 | | | 0.684 |
| AA + AG vs. GG | | FEM | | 1.00 | | [0.93; 1.08] | | 0.09 | | 0.9289 | | 22.91 | | 15 | | 0.0860 | | 0.0138 | | 34.50% | | 0.0423 | | 0.058 | | | 0.026 |
| AG vs. AA + GG | | FEM | | 0.98 | | [0.91; 1.05] | | -0.67 | | 0.5025 | | 21.88 | | 15 | | 0.1111 | | 0.0110 | | 31.40% | | 0.0523 | | 0.048 | | | 0.0500 |
| A vs. G | | FEM | | 0.99 | | [0.94; 1.04] | | -0.36 | | 0.6984 | | 24.37 | | 15 | | 0.0591 | | 0.0080 | | 38.40% | | 0.0461 | | 0.047 | | | 0.043 |
| **Cancer Type = Blood Cancers** | |  | | **Summary Measures** | | | | | | | | **Heterogeneity** | | | | | | | | | | **GoF test (p-vall)** | | | | | |
|  | | Model | | OR | | 95% C.I. | | Z-val | | p-vall | | Q-test | | d.f | | p-val | | Tau^2^ | | I^2 | | AD | | CvM | | | SW |
| AA vs. GG | | FEM | | 0.97 | | [0.83; 1.13] | | -0.34 | | 0.7353 | | - | | 0 | | - | | 0.0000 | | 0.00% | | - | | - | | | - |
| AA vs. AG + GG | | FEM | | 0.97 | | [0.84; 1.12] | | -0.43 | | 0.6699 | | 0.00 | | 0 | | - | | 0.0000 | | 0.00% | | - | | - | | | - |
| AA + AG vs. GG | | REM | | 1.01 | | [0.47; 6.86] | | 0.86 | | 0.8045 | | 6.66 | | 1 | | 0.0099 | | 0.8110 | | 85.00% | | - | | - | | | - |
| AG vs. AA + GG | | REM | | 0.97 | | [0.88; 1.07] | | -0.56 | | 0.5815 | | 6.50 | | 1 | | 0.0108 | | 0.7882 | | 84.60% | | - | | - | | | - |
| A vs. G | | REM | | 1.75 | | [0.48; 6.46] | | 0.84 | | 0.3981 | | 6.56 | | 1 | | 0.0104 | | 0.7679 | | 84.80% | | - | | - | | | - |
| **Cancer Type = Breast Cancers** | |  | | **Summary Measures** | | | | | | | | **Heterogeneity** | | | | | | | | | | **GoF test (p-vall)** | | | | | |
|  | | Model | | OR | | 95% C.I. | | Z-val | | p-vall | | Q-test | | d.f | | p-val | | Tau^2^ | | I^2 | | AD | | CvM | | | SW |
| AA vs. GG | | FEM | | 1.11 | | [0.63; 1.92] | | 0.37 | | 0.7097 | | 0.75 | | 2 | | 0.6853 | | 0.0000 | | 0.00% | | - | | - | | | - |
| AA vs. AG + GG | | FEM | | 1.07 | | [0.64; 1.79] | | 0.26 | | 0.7980 | | 0.46 | | 2 | | 0.7932 | | 0.0000 | | 0.00% | | - | | - | | | - |
| AA + AG vs. GG | | FEM | | 1.24 | | [0.82; 1.88] | | 1.75 | | 0.0800 | | 3.23 | | 2 | | 0.1989 | | 0.0507 | | 38.10% | | - | | - | | | - |
| AG vs. AA + GG | | FEM | | 0.79 | | [0.59; 1.06] | | -1.56 | | 0.1176 | | 3.12 | | 2 | | 0.2097 | | 0.0467 | | 36.00% | | - | | - | | | - |
| A vs. G | | FEM | | 1.20 | | [0.95; 1.52] | | 1.56 | | 0.1221 | | 3.28 | | 2 | | 0.1942 | | 0.0342 | | 39.00% | | - | | - | | | - |
| **Cancer Type = Cervical Cancers** | |  | | **Summary Measures** | | | | | | | | **Heterogeneity** | | | | | | | | | | **GoF test (p-vall)** | | | | | |
|  | | Model | | OR | | 95% C.I. | | Z-val | | p-vall | | Q-test | | d.f | | p-val | | Tau^2^ | | I^2 | | AD | | CvM | | | SW |
| AA vs. GG | | FEM | | 0.79 | | [0.63; 0.98] | | -2.06 | | 0.0390 | | 1.26 | | 1 | | 0.2608 | | 0.0466 | | 20.80% | | - | | - | | | - |
| AA vs. AG + GG | | FEM | | 0.82 | | [0.68; 1.00] | | -1.98 | | 0.0474 | | 2.02 | | 1 | | 0.1552 | | 0.1750 | | 50.50% | | - | | - | | | - |
| AA + AG vs. GG | | FEM | | 0.93 | | [0.75; 1.23] | | -0.85 | | 0.3969 | | 1.31 | | 1 | | 0.2515 | | 0.0121 | | 23.90% | | - | | - | | | - |
| AG vs. AA + GG | | FEM | | 0.94 | | [0.80; 1.09] | | -0.79 | | 0.4312 | | 3.20 | | 1 | | 0.0737 | | 0.0953 | | 69.70% | | - | | - | | | - |
| A vs. G | | FEM | | 0.91 | | [0.82; 1.02] | | -1.69 | | 0.0667 | | 0.09 | | 1 | | 0.0000 | | 0.0000 | | 0.00% | | - | | - | | | - |
| **Cancer Type = Colon Cancers** | |  | | **Summary Measures** | | | | | | | | **Heterogeneity** | | | | | | | | | | **GoF test (p-vall)** | | | | | |
|  | | Model | | OR | | 95% C.I. | | Z-val | | p-vall | | Q-test | | d.f | | p-val | | Tau^2^ | | I^2 | | AD | | CvM | | | SW |
| AA vs. GG | | FEM | | 0.84 | | [ 0.60; 1.20] | | -0.92 | | 0.3674 | | 2.52 | | 2 | | 0.2841 | | 0.0270 | | 20.50% | | - | | - | | | - |
| AA vs. AG + GG | | FEM | | 0.86 | | [0.64; 1.18] | | -0.90 | | 0.3700 | | 3.17 | | 2 | | 0.2049 | | 0.0484 | | 36.90% | | - | | - | | | - |
| AA + AG vs. GG | | FEM | | 0.93 | | [0.72; 1.20] | | -0.54 | | 0.5885 | | 0.40 | | 2 | | 0.8178 | | 0.0000 | | 0.00% | | - | | - | | | - |
| AG vs. AA + GG | | FEM | | 0.97 | | [0.77; 1.24] | | -0.19 | | 0.8488 | | 0.83 | | 2 | | 0.6610 | | 0.0000 | | 0.00% | | - | | - | | | - |
| A vs. G | | FEM | | 0.93 | | [0.78; 1.10] | | -0.86 | | 0.3887 | | 1.80 | | 2 | | 0.4059 | | 0.0000 | | 0.00% | | - | | - | | | - |
| **Cancer Type = Prostate Cancers** | |  | | **Summary Measures** | | | | | | | | **Heterogeneity** | | | | | | | | | | **GoF test (p-vall)** | | | | | |
|  | | Model | | OR | | 95% C.I. | | Z-val | | p-vall | | Q-test | | d.f | | p-val | | Tau^2^ | | I^2 | | AD | | CvM | | | SW |
| AA vs. GG | | FEM | | 0.97 | | [0.71; 1.31] | | -0.22 | | 0.8243 | | 0.73 | | 1 | | 0.3914 | | 0.0000 | | 0.00% | | - | | - | | | - |
| AA vs. AG + GG | | FEM | | 1.07 | | [0.81; 1.42] | | 0.50 | | 0.6183 | | 1.22 | | 1 | | 0.2686 | | 0.0125 | | 18.30% | | - | | - | | | - |
| AA + AG vs. GG | | FEM | | 0.87 | | [0.72; 1.05] | | -1.37 | | 0.1708 | | 0.00 | | 1 | | 0.9582 | | 0.0000 | | 0.00% | | - | | - | | | - |
| AG vs. AA + GG | | FEM | | 1.17 | | [0.97; 1.41] | | 1.67 | | 0.0944 | | 0.55 | | 1 | | 0.4597 | | 0.0000 | | 0.00% | | - | | - | | | - |
| A vs. G | | FEM | | 0.95 | | [0.83; 1.09] | | -0.74 | | 0.4591 | | 0.40 | | 1 | | 0.5270 | | 0.0000 | | 0.00% | | - | | - | | | - |
| **Cancer Type = Stomach Cancers** | |  | | **Summary Measures** | | | | | | | | **Heterogeneity** | | | | | | | | | | **GoF test (p-vall)** | | | | | |
|  | | Model | | OR | | 95% C.I. | | Z-val | | p-vall | | Q-test | | d.f | | p-val | | Tau^2^ | | I^2 | | AD | | CvM | | | SW |
| AA vs. GG | | FEM | | 1.71 | | [0.54; 5.34] | | 0.92 | | 0.3551 | | 0.71 | | 1 | | 0.3945 | | 0.0000 | | 0.00% | | - | | - | | | - |
| AA vs. AG + GG | | FEM | | 1.83 | | [0.60; 5.57] | | 1.07 | | 0.2846 | | 0.61 | | 1 | | 0.4333 | | 0.0000 | | 0.00% | | - | | - | | | - |
| AA + AG vs. GG | | FEM | | 0.98 | | [0.54; 1.76] | | -0.06 | | 0.9657 | | 0.49 | | 1 | | 0.4856 | | 0.0000 | | 0.00% | | - | | - | | | - |
| AG vs. AA + GG | | FEM | | 1.21 | | [0.67; 2.20] | | 0.63 | | 0.5301 | | 0.15 | | 1 | | 0.7015 | | 0.0000 | | 0.00% | | - | | - | | | - |
| A vs. G | | FEM | | 1.10 | | [0.69; 1.76] | | 0.41 | | 0.6853 | | 0.80 | | 1 | | 0.3714 | | 0.0000 | | 0.00% | | - | | - | | | - |
| **Cancer Type = Other Cancers** | |  | | **Summary Measures** | | | | | | | | **Heterogeneity** | | | | | | | | | | **GoF test (p-vall)** | | | | | |
|  | | Model | | OR | | 95% C.I. | | Z-val | | p-vall | | Q-test | | d.f | | p-val | | Tau^2^ | | I^2 | | AD | | CvM | | | SW |
| AA vs. GG | | FEM | | 1.36 | | [0.96; 1.92] | | 1.78 | | 0.0744 | | 0.80 | | 1 | | 0.3721 | | 0.0000 | | 0.00% | | - | | - | | | - |
| AA vs. AG + GG | | FEM | | 1.20 | | [0.90; 1.60] | | 1.25 | | 0.2122 | | 0.20 | | 1 | | 0.6580 | | 0.0000 | | 0.00% | | - | | - | | | - |
| AA + AG vs. GG | | FEM | | 1.29 | | [0.96; 1.73] | | 1.94 | | 0.0524 | | 1.23 | | 1 | | 0.2667 | | 0.0087 | | 18.90% | | - | | - | | | - |
| AG vs. AA + GG | | FEM | | 0.91 | | [0.71; 1.16] | | -0.75 | | 0.4556 | | 0.92 | | 1 | | 0.3368 | | 0.0000 | | 0.00% | | - | | - | | | - |
| A vs. G | | FEM | | 1.19 | | [1.00; 1.41] | | 2.00 | | 0.0450 | | 1.01 | | 1 | | 0.3158 | | 0.0001 | | 0.00% | | - | | - | | | - |
| **Ethnicity = African** | |  | | **Summary Measures** | | | | | | | | **Heterogeneity** | | | | | | | | | | **GoF test (p-vall)** | | | | | |
|  | | Model | | OR | | 95% C.I. | | Z-val | | p-vall | | Q-test | | d.f | | p-val | | Tau^2^ | | I^2 | | AD | | CvM | | | SW |
| AA vs. GG | | FEM | | 0.80 | | [0.37; 1.70] | | -0.59 | | 0.5600 | | 2.80 | | 1 | | 0.0943 | | 0.6406 | | 64.30% | | - | | - | | | - |
| AA vs. AG + GG | | FEM | | 0.69 | | [0.32; 1.46] | | -0.97 | | 0.3323 | | 2.79 | | 1 | | 0.0947 | | 0.6296 | | 64.20% | | - | | - | | | - |
| AA + AG vs. GG | | FEM | | 1.48 | | [1.08; 2.03] | | 2.47 | | 0.0135 | | 0.72 | | 1 | | 0.3964 | | 0.0000 | | 0.00% | | - | | - | | | - |
| AG vs. AA + GG | | FEM | | 0.61 | | [0.44; 0.84] | | -2.98 | | 0.0010 | | 0.04 | | 1 | | 0.8358 | | 0.0000 | | 0.00% | | - | | - | | | - |
| A vs. G | | FEM | | 1.28 | | [0.98; 1.67] | | 1.78 | | 0.0191 | | 2.49 | | 1 | | 0.0000 | | 0.0603 | | 59.80% | | - | | - | | | - |
| **Ethnicity = Asian** | |  | | **Summary Measures** | | | | | | | | **Heterogeneity** | | | | | | | | | | **GoF test (p-vall)** | | | | | |
|  | | Model | | OR | | 95% C.I. | | Z-val | | p-vall | | Q-test | | d.f | | p-val | | Tau^2^ | | I^2 | | AD | | CvM | | | SW |
| AA vs. GG | | FEM | | 0.61 | | [0.02; 15.10] | | -0.30 | | 0.7600 | | - | | 0 | | - | | 0.0000 | | 0.00% | | - | | - | | | - |
| AA vs. AG + GG | | FEM | | 0.62 | | [0.02; 15.24] | | -0.30 | | 0.7669 | | - | | 0 | | - | | 0.0000 | | 0.00% | | - | | - | | | - |
| AA + AG vs. GG | | FEM | | 2.11 | | [0.91; 4.85] | | 1.75 | | 0.0602 | | 3.54 | | 1 | | 0.0598 | | 0.9104 | | 71.80% | | - | | - | | | - |
| AG vs. AA + GG | | REM | | 0.56 | | [0.11; 2.89] | | -0.70 | | 0.0800 | | 3.91 | | 1 | | 0.0480 | | 1.0534 | | 74.40% | | - | | - | | | - |
| A vs. G | | FEM | | 2.11 | | [0.93; 4.81] | | 1.78 | | 0.0753 | | 3.10 | | 1 | | 0.0785 | | 0.7258 | | 67.70% | | - | | - | | | - |
| **Ethnicity = Caucasian** | |  | | **Summary Measures** | | | | | | | | **Heterogeneity** | | | | | | | | | | **GoF test (p-vall)** | | | | | |
|  | | Model | | OR | | 95% C.I. | | Z-val | | p-vall | | Q-test | | d.f | | p-val | | Tau^2^ | | I^2 | | AD | | CvM | | | SW |
| AA vs. GG | | REM | | 0.97 | | [0.85; 1.16] | | -0.53 | | 0.5965 | | 1.06 | | 10 | | 0.2965 | | 0.0072 | | 15.50% | | 0.3506 | | 0.351 | | | 0.357 |
| AA vs. AG + GG | | REM | | 0.97 | | [0.87; 1.08] | | -0.57 | | 0.5689 | | 10.67 | | 10 | | 0.3834 | | 0.0021 | | 6.30% | | 0.3204 | | 0.312 | | | 0.339 |
| AA + AG vs. GG | | REM | | 0.98 | | [0.91; 1.05] | | -0.63 | | 0.5254 | | 8.81 | | 10 | | 0.5500 | | 0.0000 | | 0.00% | | 0.2659 | | 0.297 | | | 0.234 |
| AG vs. AA + GG | | REM | | 1.00 | | [0.93; 1.07] | | -0.05 | | 0.9640 | | 5.85 | | 10 | | 0.8273 | | 0.0000 | | 0.00% | | 0.3328 | | 0.302 | | | 0.441 |
| A vs. G | | REM | | 0.97 | | [0.87; 1.08] | | -0.57 | | 0.5689 | | 11.78 | | 10 | | 0.3003 | | 0.0016 | | 15.10% | | 0.4644 | | 0.433 | | | 0.4900 |
| **Ethnicity = Mixed** | |  | | **Summary Measures** | | | | | | | | **Heterogeneity** | | | | | | | | | | **GoF test (p-vall)** | | | | | |
|  | | Model | | OR | | 95% C.I. | | Z-val | | p-vall | | Q-test | | d.f | | p-val | | Tau^2^ | | I^2 | | AD | | CvM | | | SW |
| AA vs. GG | | FEM | | 1.29 | | [0.36; 4.67] | | 0.40 | | 0.6900 | | 4.67 | | 0 | | 0.0000 | | 0.0000 | | 0.00% | | - | | - | | | - |
| AA vs. AG + GG | | FEM | | 1.44 | | [0.42; 4.96] | | 0.57 | | 0.5672 | | - | | 0 | | - | | 0.0000 | | 0.00% | | - | | - | | | - |
| AA + AG vs. GG | | FEM | | 0.86 | | [0.43; 1.72] | | -0.42 | | 0.6774 | | 0.00 | | 0 | | - | | 0.0000 | | 0.00% | | - | | - | | | - |
| AG vs. AA + GG | | FEM | | 1.30 | | [0.65; 2.62] | | 0.73 | | 0.4600 | | 0.00 | | 0 | | - | | 0.0000 | | 0.00% | | - | | - | | | - |
| A vs. G | | FEM | | 0.98 | | [0.57; 1.04] | | -0.08 | | 0.9343 | | 0.00 | | 0 | | - | | 0.0000 | | 0.00% | | - | | - | | | - |
