# Supplementary material for: Statistical meta-analysis to investigate the association between the Interleukin-6 (IL-6) gene polymorphisms and cancer risk
Source: PLoS One. 2021 Mar 8;16(3):e0247055. doi: 10.1371/journal.pone.0247055 (PMC7939379; doi:10.1371/journal.pone.0247055)
Supplement: S1 File — (DOCX) [file pone.0247055.s002.docx]

**Forest plot**

In the forest plot the square of the horizontal line represent the individual study-specific odds ratios (ORs) with 95% confidence intervals (CIs) and the black area of the squares represent the corresponding study weight. The black diamond reflects the pooled OR and the lateral points of the diamond represent the CI of the overall analyses. The solid vertical lines are the OR of 1 which is line of no effect. The dashed vertical line shows the corresponding pooled OR of the analyses.


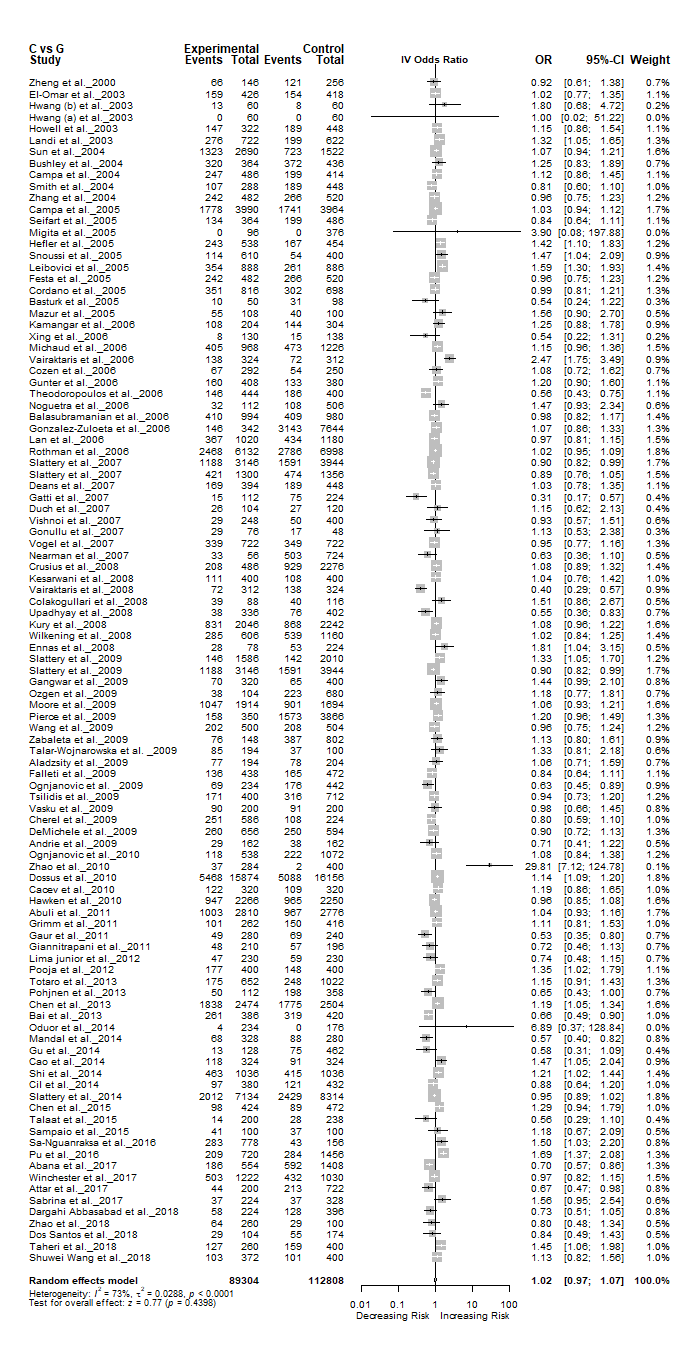


**Fig S1 A.** **Forest plot of IL-6 -174G/C polymorphisms for overall cancer using allelic model (C vs. G).**

**
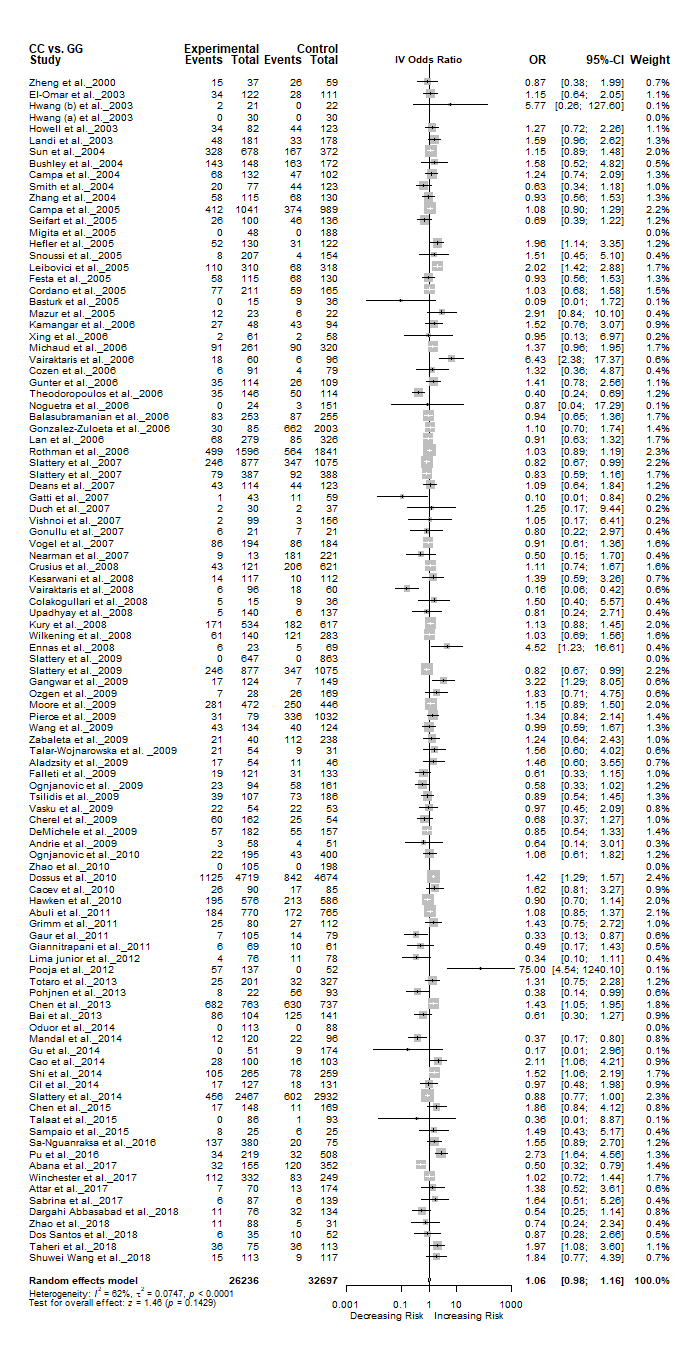
**

**Fig S1 B. Forest plot for CC vs. GG of IL-6 -174G/C polymorphism with overall cancer risk.**


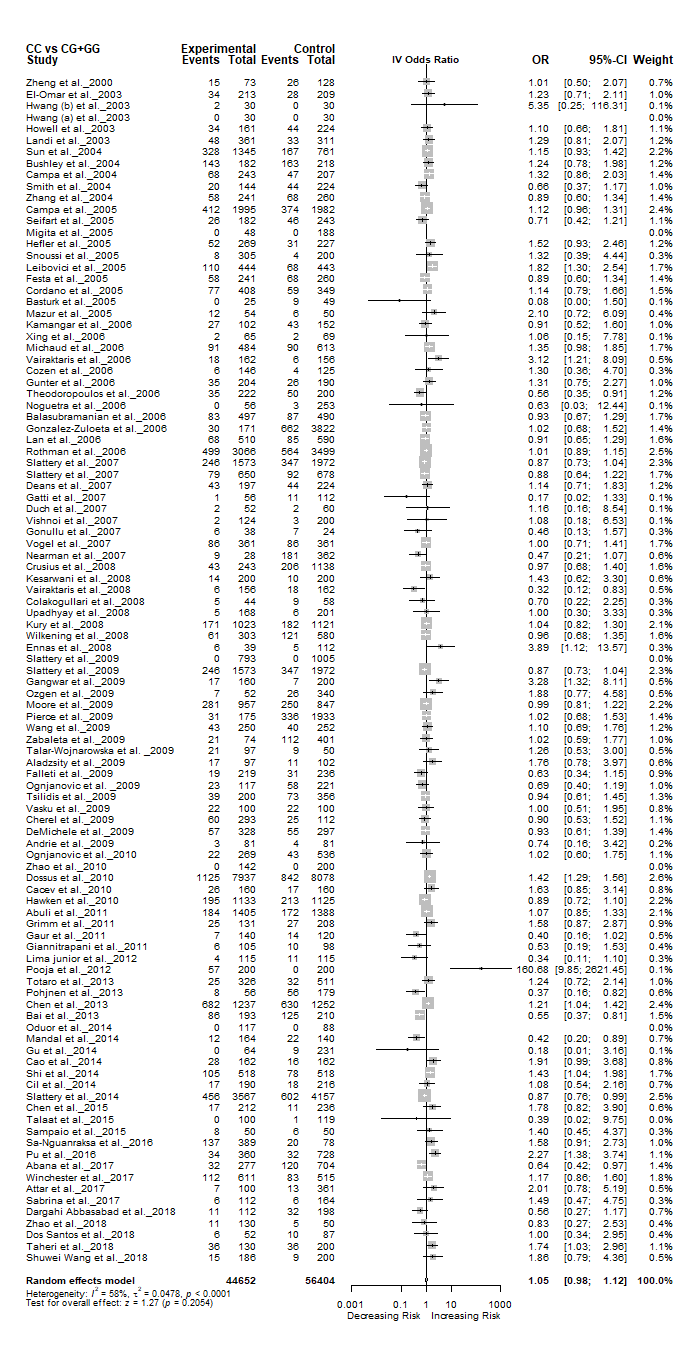


**Fig S1 C. Forest plot for CC vs. CG + GG of IL-6 -174G/C polymorphism with overall cancer risk.**

**
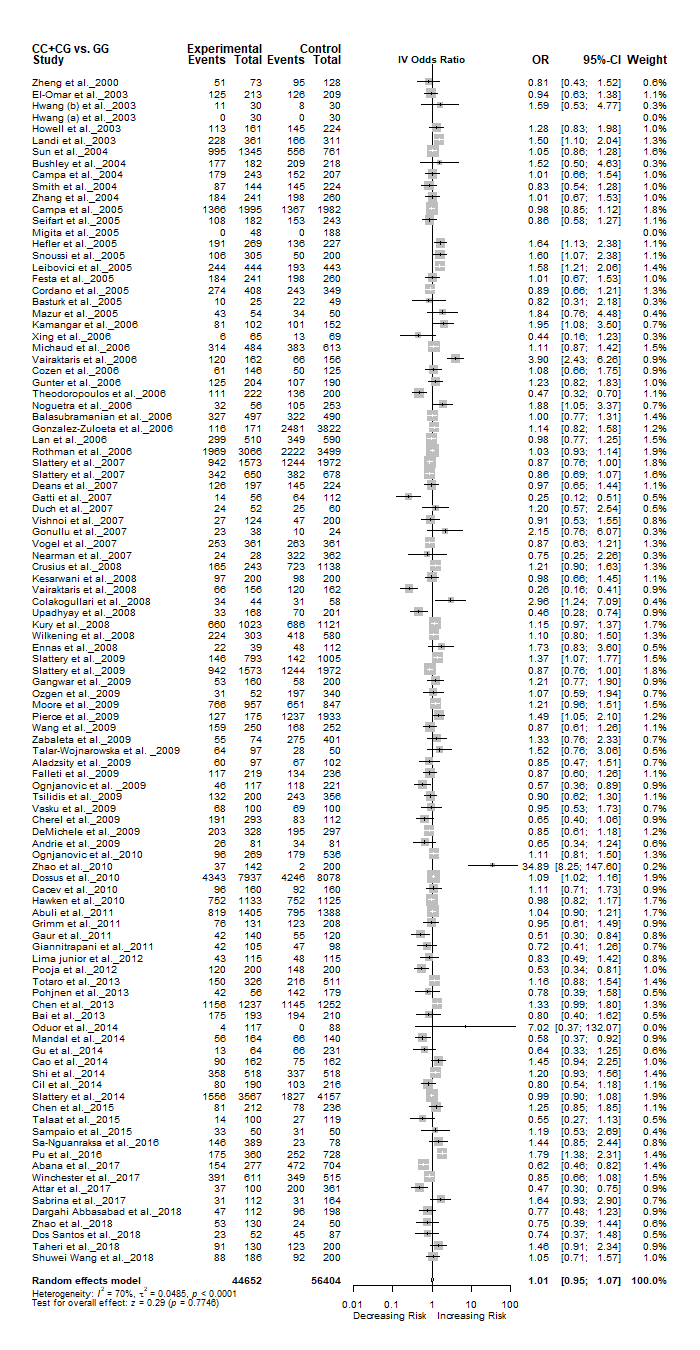
**

**Fig S1 D. Forest plot for CC + CG vs. GG of IL-6 -174G/C polymorphism with overall cancer risk.**


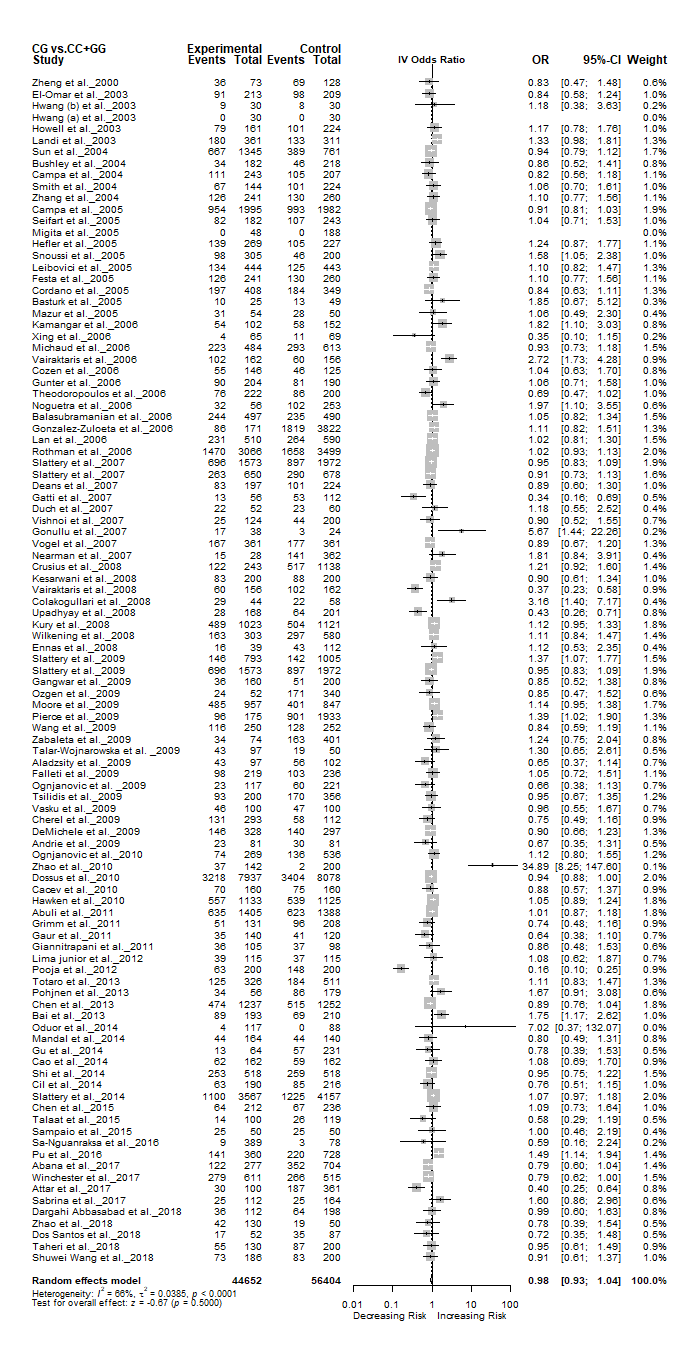


**Fig S1 E. Forest plot for CG vs. CC + GG of IL-6 -174G/C polymorphism with overall cancer risk.**


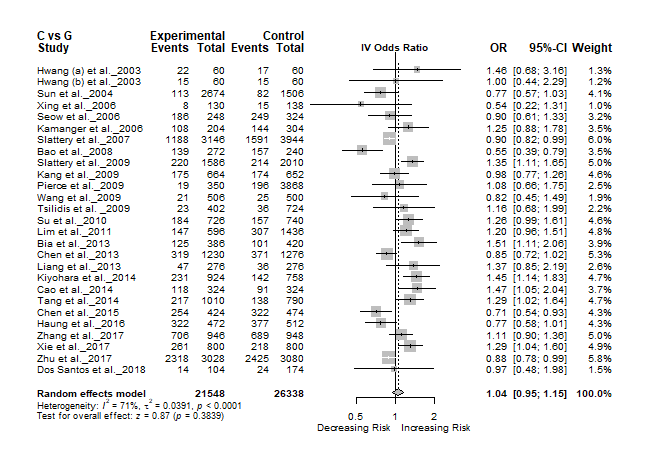


**Fig S1 F. Forest plot of IL-6-572G/C polymorphisms for overall cancer using allelic model (C vs. G).**


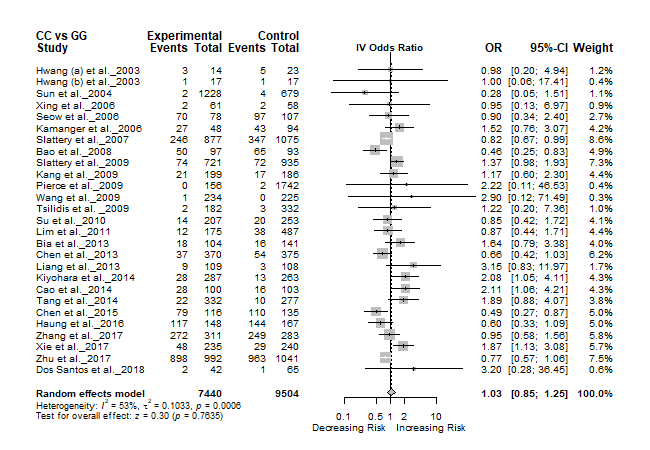


**Fig S1 G. Forest plot for CC vs. GG of IL-6 -572G/C polymorphism with overall cancer risk.**


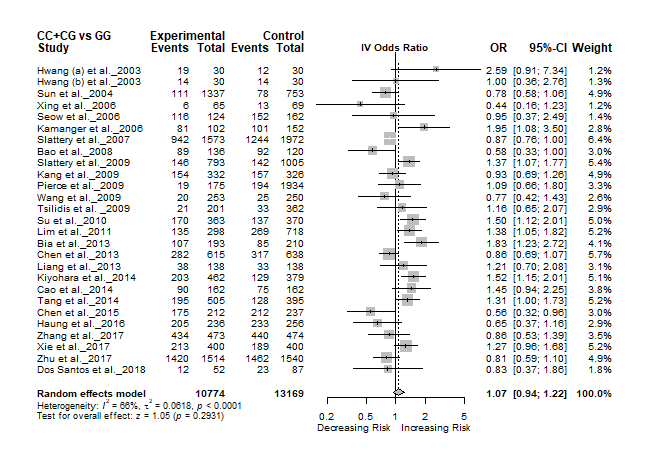


**Fig S1 H. Forest plot for CC + CG vs. GG of IL-6 -572G/C polymorphism with overall cancer risk.**


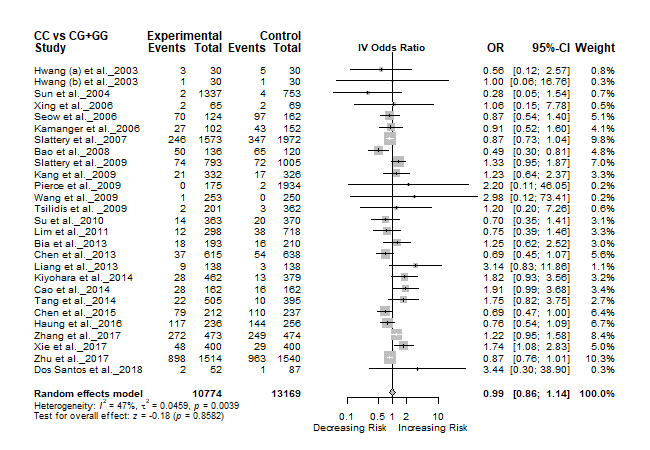


**Fig S1 I. Forest plot for CC vs. CG + GG of IL-6 -572G/C polymorphism with overall cancer risk.**


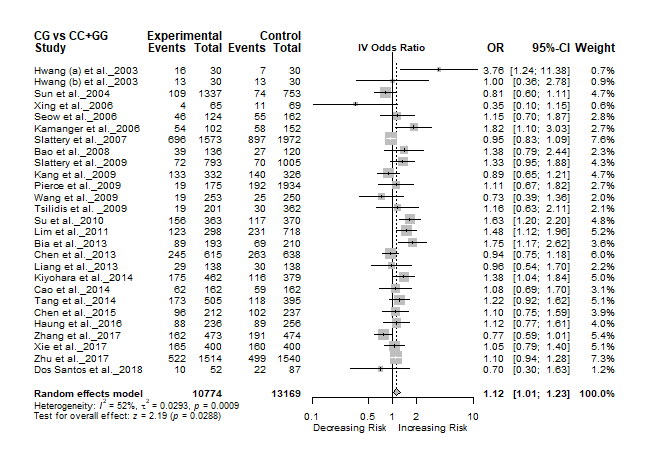


**Fig S1 J. Forest plot for CG vs. CC + GG of IL-6 -572G/C polymorphism with overall cancer risk.**


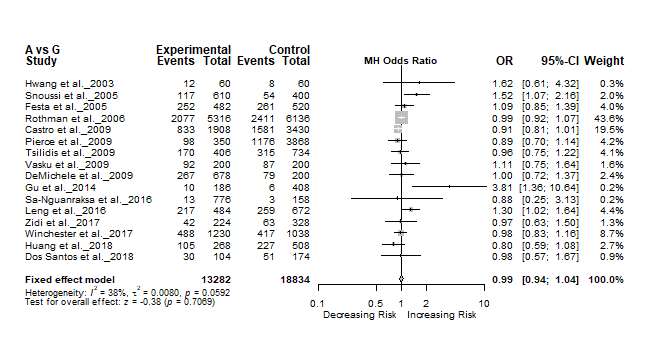


**Fig S1 K. Forest plot of IL-6 -597G/A polymorphism for overall cancer using allelic model (A vs. G)**


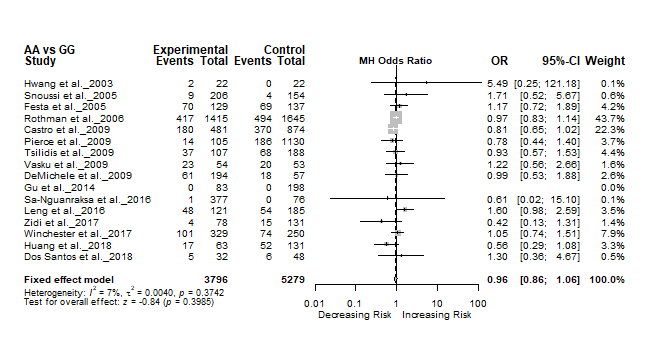


**Fig S1 L. Forest plot for AA vs. GG of IL-6 -597G/A polymorphism with overall cancer risk.**

**
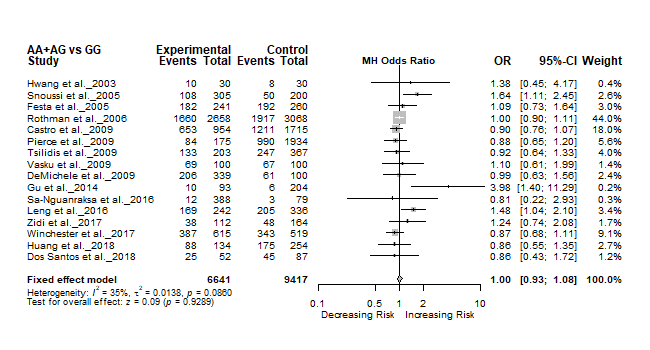
**

**Fig S1 M. Forest plot for AA + AG vs. GG of IL-6 -597G/A polymorphism with overall cancer risk.**


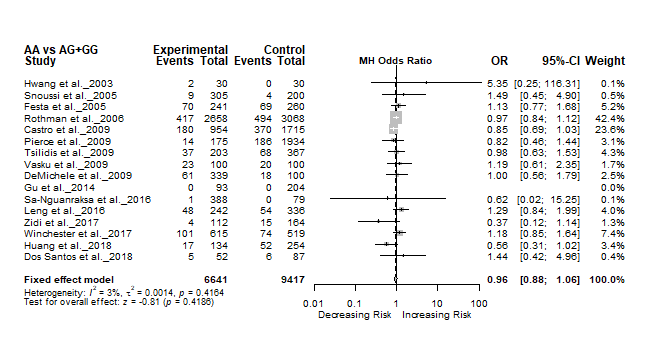


**Fig S1 N. Forest plot for AA vs. AG + GG of IL-6 -597G/A polymorphism with overall cancer risk.**


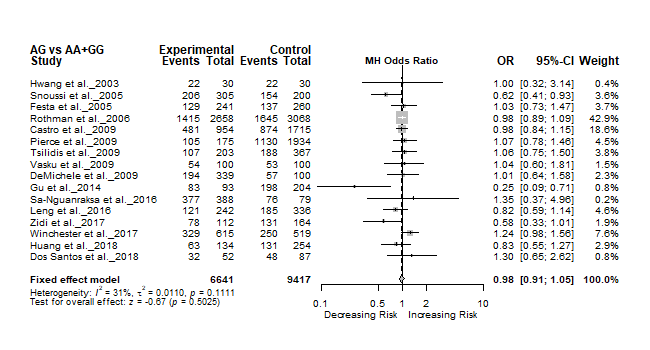


**Fig S1 O. Forest plot for AA vs. AG + GG of IL-6 -597G/A polymorphism with overall cancer risk.**
