# Supplementary material for: Statistical meta-analysis to investigate the association between the Interleukin-6 (IL-6) gene polymorphisms and cancer risk
Source: PLoS One. 2021 Mar 8;16(3):e0247055. doi: 10.1371/journal.pone.0247055 (PMC7939379; doi:10.1371/journal.pone.0247055)
Supplement: S2 File — (DOCX) [file pone.0247055.s003.docx]

**Publication Bias**

For testing the publication bias we used the following test:

**Egger’s test:**

The Egger’s test is perform the following hypothesis testing by p-value

H_0_ (Null hypothesis) : Symmetry in the funnel plot vs.

H_1_ (Alternative hypothesis) : Asymmetry in the funnel plot

If p-value ≤ 0.05we reject Null hypothesis (H_0_)

If p-value > 0.05 we accept the null hypothesis (H_0_) and reject the alternative hypothesis (H_1_).

**C vs. G: (rs1800795 or -174G/C)**

Linear regression test of funnel plot asymmetry

Data: C vs. G meta data

t = -0.71259, df = 99, p-value = 0.4778

Alternative hypothesis: asymmetry in funnel plot

Sample estimates: bias se.bias slope

-0.22779871 0.31967744 0.05190845

**Fig S2 A. Egger’s linear regression test for C vs. G of IL-6 -174G/C polymorphism**

**C vs. G: (rs1800796 or -572G/C)**

Linear regression test of funnel plot asymmetry

Data: C vs. G Meta data

t = 1.0003, df = 25, p-value = 0.3267

Alternative hypothesis: asymmetry in funnel plot

Sample estimates: bias se.bias slope

0.72301424 0.72278894 -0.07044322

**Fig S2 B. Egger’s linear regression test for C vs. G of IL-6 -572G/C polymorphism**

**A vs. G (rs1800797 or -597A/G)**

Linear regression test of funnel plot asymmetry

Data: A vs. G Meta data

t = 1.6682, df = 14, p-value = 0.1175

Alternative hypothesis: asymmetry in funnel plot

Sample estimates: bias se.bias slope

0.81375987 0.48781244 -0.07446294

**Fig S2 C. Egger’s linear regression test for A vs. G of IL-6 597A/G polymorphism**

**Table S2 A. Publication bias outcome for IL-6 gene polymorphisms**

| **Polymorphisms** | **Begg's test** | | **Egger's test** | | |
| --- | --- | --- | --- | --- | --- |
|  | **z-value** | **p-value** | **t-value** | **d.f.** | **p-value** |
| **rs-1800795 (172G/C)** |  |  |  |  |  |
| CC vs. GG | 0.3315 | 0.7403 | -0.5750 | 96 | 0.5667 |
| C vs. G | -0.2494 | 0.8030 | -0.7126 | 99 | 0.4778 |
|  |  |  |  |  |  |
| **rs-1800796 (572(G/C)** |  |  |  |  |  |
| CC vs. GG | 1.1883 | 0.2347 | 1.4254 | 25 | 0.1664 |
| C vs. G | -0.5212 | 0.6022 | 1.0003 | 25 | 0.3267 |
|  |  |  |  |  |  |
| **rs-1800797 (597A/G)** |  |  |  |  |  |
| AA vs.GG | 0.3464 | 0.7290 | 0.5352 | 13 | 0.6016 |
| A vs. G | 1.3507 | 0.1768 | 1.6682 | 14 | 0.1175 |

**Table S2 B. Examining the Influence of Outcome Reporting Bias**

| **Polymorphisms** | **Begg's test** | | **Egger's test** | | |
| --- | --- | --- | --- | --- | --- |
|  | **p-value** | **comment** |  | **p-value** | **comment** |
| **rs-1800795** |  |  | **rs-1800795** |  |  |
| CC vs. GG | p-value = 0.7403 | No publication bias | CC vs. GG | p-value = 0.5667 | No publication bias |
| C vs. G | p-value = 0.803 | No publication bias | C vs. G | p-value = 0.4778 | No publication bias |
|  |  |  |  |  |  |
| **rs-1800796** |  |  | **rs-1800796** |  |  |
| CC vs. GG | p-value = 0.2347 | No publication bias | CC vs. GG | p-value = 0.1664 | No publication bias |
| C vs. G | p-value = 0.6022 | No publication bias | C vs. G | p-value = 0.3267 | No publication bias |
|  |  |  |  |  |  |
| **rs-1800797** |  |  | **rs-1800797** |  |  |
| AA vs. GG | p-value = 0.729 | No publication bias | AA vs.GG | p-value = 0.6016 | No publication bias |
| A vs. G | p-value = 0.1768 | No publication bias | A vs. G | p-value = 0.1175 | No publication bias |

**Sensitivity Analysis Results**

**Table S2 C. Sensitivity analysis for IL-6 gene rs1800795 polymorphism of overall cancer**

|  |  |  | **CC vs. GG** | | **CC vs. CG + GG** | | **CC + CG vs. GG** | | **CG vs. CC + GG** | | **C vs. G** | |
| --- | --- | --- | --- | --- | --- | --- | --- | --- | --- | --- | --- | --- |
|  |  |  | **Effect Estimate** | | **Effect Estimate** | | **Effect Estimate** | | **Effect Estimate** | | **Effect Estimate** | |
|  | Study  Number | Sample  size | OR (95% CI) | p-value | OR (95% CI) | p-value | OR (95% CI) | p-value | OR (95% CI) | p-value | OR (95% CI) | p-value |
| **Overall** | 103 | 102493 | 1.06 [0.98; 1.16] | 0.1429 | 1.05 [0.98; 1.12] | 0.2054 | 1.02 [0.99; 1.05] | 0.0615 | 0.99 [0.96; 1.01] | 0.2681 | 1.02 [0.97; 1.06] | 0.4459 |
| **With HWE**  **satisfaction** | 78 | 60997 | 1.08 [0.99; 1.18] | 0.0874 | 1.05 [0.98; 1.13] | 0.1380 | 1.02 [0.95; 1.09] | 0.5965 | 1.00 [0.94; 1.05] | 0.8827 | 1.02 [0.97; 1.07] | 0.4615 |

**Table S2 D. Sensitivity analysis for IL-6 gene rs1800796 polymorphism of overall cancer**

|  |  |  | **CC vs. GG** | | **CC vs. CG + GG** | | **CC + CG vs. GG** | | **CG vs. CC + GG** | | **C vs. G** | |
| --- | --- | --- | --- | --- | --- | --- | --- | --- | --- | --- | --- | --- |
|  |  |  | **Effect Estimate** | | **Effect Estimate** | | **Effect Estimate** | | **Effect Estimate** | | **Effect Estimate** | |
|  | Study  Number | Sample  size | OR (95% CI) | p-value | OR (95% CI) | p-value | OR (95% CI) | p-value | OR (95% CI) | p-value | OR (95% CI) | p-value |
| **Overall** | 27 | 24138 | 1.03 [0.85; 1.25] | 0.7635 | 0.99 [0.86; 1.14] | 0.8582 | 1.07 [0.94; 1.22] | 0.2931 | 1.12 [1.01; 1.23] | 0.0288 | 1.04 [0.95; 1.15] | 0.3839 |
| **Overall with HWE** | 21 | 17498 | 1.07 [0.84; 1.36] | 0.5905 | 1.04 [0.87; 1.23] | 0.6932 | 1.09 [0.97; 1.21] | 0.4544 | 1.09 [0.97; 1.21] | 0.1384 | 1.05 [0.94; 1.17] | 0.3675 |

**Table S2 E. Sensitivity analysis for IL-6 gene rs1800797 polymorphism of overall cancer**

|  |  |  | **AA vs. GG** | | **AA vs. AG + GG** | | **AA + AG vs. GG** | | **AG vs. AA + GG** | | **A vs. G** | |
| --- | --- | --- | --- | --- | --- | --- | --- | --- | --- | --- | --- | --- |
|  |  |  | **Effect Estimate** | | **Effect Estimate** | | **Effect Estimate** | | **Effect Estimate** | | **Effect Estimate** | |
|  | Study  Number | Sample  size | OR (95% CI) | p-value | OR (95% CI) | p-value | OR (95% CI) | p-value | OR (95% CI) | p-value | OR (95% CI) | p-value |
| **Overall** | 16 | 16167 | 0.96 [0.85; 1.08] | 0.5152 | 0.97 [0.87; 1.07] | 0.5064 | 1.00 [0.93; 1.08] | 0.9289 | 0.98 [0.91; 1.05] | 0.5025 | 0.99 [0.94; 1.04] | 0.7169 |
| **Overall with HWE** | 15 | 15891 | 0.96 [0.87; 1.07] | 0.4932 | 0.97 [0.88; 1.06] | 0.5245 | 1.00 [0.93; 1.07] | 0.9812 | 0.98 [0.92; 1.05] | 0.6624 | 1.02 [0.93; 1.11] | 0.6984 |
