# Supplementary material for: Statistical meta-analysis to investigate the association between the Interleukin-6 (IL-6) gene polymorphisms and cancer risk
Source: PLoS One. 2021 Mar 8;16(3):e0247055. doi: 10.1371/journal.pone.0247055 (PMC7939379; doi:10.1371/journal.pone.0247055)
Supplement: Attachment — Submitted filename: Response_to_Editor_PONE-D-20-23035.docx [file pone.0247055.s008.docx]

**General comment:** We note that the manuscript is reporting a meta-analysis on genetic association studies. Before we can further progress with your submission, we need you to provide us with additional information in relation to this meta-analysis.

Please provide a response to each of the points listed below in the Comments section of the submission form in Editorial Manager and complete any necessary revisions to the manuscript to ensure that each of the points is adequately discussed in the text of the submission:

**Author’s response:** Thanks for your important suggestion. We revised our manuscript accordingly.

**Specific comment-1:** Please describe the rationale for carrying out this meta-analysis in the context of current knowledge in the field. Please indicate whether there are previous meta-analyses in the literature which covered this research question (or closely related ones) and if that is the case, list the relevant reference(s) and comment on how the current meta-analysis differs from the previously published reports.

**Author’s response:** Thanks for these important queries. We revised the introduction section to clear these queries. Please see the last paragraph of introduction section to get the answer (page-3).

**Specific comment-2:** Please provide the full details of the databases searched to identify the studies included in this meta-analysis.

**Author’s response:** Thanks for this important suggestion. The step wise database searches to identify the relevant studies in our meta-analysis are discussed in **materials and methods sectio**n of main manuscript (page-3; subsections: **Search strategy, Eligibility Criteria, Data extraction**). Also, the **Fig. 1** showed the study selection process at a glance. The datasets are given in supporting files (Data S1-S3). The metadata were collected through online database search. We pooled together the relevant study by used some keywords from online database portal PubMed, PubMed Central, Google Scholar and Web of Science. We investigate the study through some steps to include the meta-analysis. Firstly, we consider the title and abstract for all papers and primarily removed the irrelevant and incomplete studies. Secondly, we removed the duplicated study. Thirdly, we filtered the data with some inclusion-exclusion criteria. Fourthly, the relevant study was again removed for incomplete information. Then the study selected for this meta-analysis with needed variables. To test the validity of any selected studies for this meta-analysis, HWE test was performed using the Chi-square statistic.

**Specific comment-3:** Please clarify whether there are previous genome-wide association studies (GWAS) relevant to the research question covered by the meta-analysis and whether these were included in the analysis. If previous related GWAS are available, please indicate whether the results of this meta-analysis are consistent with the findings of the GWAS publications, and if this is not the case, please provide an explanation for this discrepancy.

**Author’s response:** Thanks for these important queries. We discussed the GWAS findings on the topic of association between gene polymorphisms and cancer risk in the second paragraph of introduction section (page-2). Again, we know the dataset of the meta-analysis was the collection of different individual GWAS publications. Each of the GWAS publications was conducted with different number of sample size, types of cancer and ethnic group. However, all the GWAS analyses were not found the similar findings; they reported both significant and insignificant association between IL-6 gene polymorphisms and cancer risk. The meta-analysis was pooled all results together from GWAS to remove the discrepancy and draw a valid and more consensus conclusion. The meta-analysis also conducts for large sample size, compare to different ethnic group with cancer risk. Therefore, the results of meta-analysis would more consistent than individual GWAS findings.
