# Supplementary material for: Statistical meta-analysis to investigate the association between the Interleukin-6 (IL-6) gene polymorphisms and cancer risk
Source: PLoS One. 2021 Mar 8;16(3):e0247055. doi: 10.1371/journal.pone.0247055 (PMC7939379; doi:10.1371/journal.pone.0247055)
Supplement: Attachment — Submitted filename: Response to Reviwers_PONE-D-20-23035.docx [file pone.0247055.s009.docx]

**Response to Review Comments**

**Review Comments:** Thank you for submitting your manuscript to PLOS ONE. After careful consideration, we feel that it has merit but does not fully meet PLOS ONE’s publication criteria as it currently stands. Therefore, we invite you to submit a revised version of the manuscript that addresses the points raised during the review process.

In the manuscript, the authors performed meta-analyses with large sample sizes. The findings, if based on appropriate statistical methods, from this study may provide us useful information in the area. However, when you apply "standard" meta-analysis models (e.g., fixed and random effect models), you need to check the fit of the models; otherwise, the outputs from inadequate models might be misleading. Please perform goodness of fit tests for your models. You can use the gof tests described in the following reference: Chen, Z., Zhang, G., & Li, J. (2015). Goodness-of-fit test for meta-analysis. Scientific reports, 5, 16983. If indeed your models are lack of fit, please revise your paper accordingly (e.g., you can follow the suggestions offered in the above mentioned reference). After receiving your revised manuscript, I will be happy to reconsider your manuscript for possible publication.

**Author’s response:** Thank you very much for your kind suggestion. We revised our manuscript accordingly. At first, we introduced the importance GoF test in the last paragraph of introduction section (page 4, highlighted portion with blue color). Then we provided the short summary of GoF test in the materials and methods section (pages 13-14, highlighted portion with blue color). We observed that in some cases GoF test based fitted models contradicts with the fitted models based on Cochran’s homogeneity test and changes the previous decision about the association between gene polymorphisms and cancer risks. In particularly, we observed the changes with some overall and subgroup cases of all polymorphisms (rs1800795, rs1800796, rs1800797). For example,

- For rs1800795, the contradictory model were observed for overall cases and Caucasian ethnic group under two genetic combinations (CC + CG vs. GG; CG vs. CC + GG); blood, liver, skin and stomach cancer under all genetic combinations (CC vs. GG; CC vs. CG + GG; CC + CG vs. GG; CG vs. CC + GG; C vs. G); breast cancer under three genetic combinations (CC vs. GG; CC vs. CG + GG; CG vs. CC + GG); colon cancer (CC vs. CG + GG), lung cancer (CC vs. GG; CC + CG vs. GG); prostate cancer (CG vs. CC + GG); Asian ethnic group under four genetic combinations (CC vs. CG + GG; CC + CG vs. GG; CG vs. CC + GG; C vs. G) (Please see additional file ‘S1 Table’, Highlighted portion with blue color). The significant association were changed in the case of liver cancer (CG vs. CC + GG) (Table 2, page 13, highlighted portion with blue color) and Asian ethnicity (CC + CG vs. GG and C vs. G) (Table 2, page 17, highlighted portion with bold & blue color).
- For rs1800796, the contradictory model were observed for the case of colon cancer under three genetic combinations (CC vs. CG + GG; CC + CG vs. GG; CG vs. CC + GG); prostate and stomach cancer for all genetic combinations (CC vs. GG; CC vs. CG + GG; CC + CG vs. GG; CG vs. CC + GG; C vs. G); Caucasian populations (CC vs. GG; CC vs. CG + GG) (Please see additional file ‘S1 Table’, Highlighted portion with blue color). The significant association were changed only the case of stomach cancer (CC + CG vs. GG) (Table 3, page-18, highlighted portion with bold & blue color).
- For rs1800797, the contradictory model were observed for the case of overall cancer under two genetic combinations (CC vs. GG; CC vs. CG + GG); and Caucasian populations under all genetic combinations (CC vs. GG; CC vs. CG + GG; CC + CG vs. GG; CG vs. CC + GG; C vs. G) (Please see additional file ‘S1 Table’, Highlighted portion with blue color). There were no significant association changes were observed (Table 3, page-19).

We also updated the discussion and conclusion section based on the revised manuscript (page 24-25, highlighted with blue color)

**Reference:** Chen, Z., Zhang, G., & Li, J. (2015). Goodness-of-fit test for meta-analysis., Scientific reports, 5, 16983)
