# Supplementary material for: Statistical meta-analysis to investigate the association between the Interleukin-6 (IL-6) gene polymorphisms and cancer risk
Source: PLoS One. 2021 Mar 8;16(3):e0247055. doi: 10.1371/journal.pone.0247055 (PMC7939379; doi:10.1371/journal.pone.0247055)
Supplement: Attachment — Submitted filename: Respone to Reviewers_PONE-D-20-23035R2.docx [file pone.0247055.s010.docx]

**Dear Professor Zhongxue Chen**

**Academic Editor, PLOS ONE**

Thank you very much for giving us the opportunity to re-revise our manuscript (ID: PONE-D-20-23035R1) according to the reviewer comments for possible publication in *PLOS ONE*. We revised our manuscript accordingly as follows:

**Reviewer #1 General Comments:** Thank you for the opportunity to review this manuscript. This is a well-conducted meta-analysis that thoroughly analyzed the associations between these three IL6 SNPs and cancer risk. I have a few minor comments:

**Minor comments-1:** This manuscript could benefit from some proofreading by a native English speaker, as there are a number of grammatical errors throughout the paper. In particular, the phrase "IL-6 gene" needs to have the word "the" preceding it, or it can be replaced by "IL6" in italics without the word "the" to refer to the gene itself.

**Author’s response:** Thanks for your kind suggestion, we tried to revise the manuscript accordingly and replaced “IL-6 gene” by the “the IL-6 gene” throughout the manuscript (Highlighted by Track changes).

**Minor comments-2:** Are the studies listed in Table 1 in any particular order within each SNP? If so, this should be included as a footnote. If not, I would recommend putting them in some order that would make it easier for the reader to review.

**Author’s response:** Yes, the studies listed in Table 1were ordered according to the year of publication for individual SNPs (Table 1, pages 5-8). Also, we added a footnote for the reader’s convenience (Table 1, page 8, Highlighted by Track changes). Thank you so much for the suggestions and going through in that detail.

**Minor comments-3:** In Tables 2-4, add footnotes indicating that the ORs for the ethnicity subgroups are for overall cancer risk. Similarly, specify in the methods/results/discussion that the models are estimating overall cancer risk within each ethnicity subgroup.

**Author’s response:** Thanks again you for your suggestions about Tables 2-4.We updated Tables 2-4 accordingly and added footnote with each table to indicate that the ORs for the ethnicity subgroups are for overall cancer risk (Table 2, page 16; Table 3, page 17; Table 4, page 18; Highlighted by Track changes). We also specified this issue in the results and discussion section of the manuscript (Pages 19, 21-24).

**Minor comments-4:** In Table 4, there is a typographical error for the OR for breast cancer in the column for CG vs. CC + GG. There is a space missing between the OR and the CI: 0.98[0.91; 1.04]

**Author’s response:** Thank you so much for your comments. This error was found in Table 2 and we updated it carefully (Table 2, page 16; Highlighted by Track changes).

With best regards,

**Prof. Md. Nurul Haque Mollah,** PhD

Head of Bioinformatics Lab.(Dry),

Department of Statistics,

University of Rajshahi, Rajshahi,

BANGLADESH.
